# On NACK-Based rDWS Algorithm for Network Coded Broadcast

**DOI:** 10.3390/e21090905

**Published:** 2019-09-17

**Authors:** Sovanjyoti Giri, Rajarshi Roy

**Affiliations:** Department of Electronics and Electrical Communication Engineering, Indian Institute of Technology, Kharagpur, West Bengal 721302, India; royr@ece.iitkgp.ernet.in

**Keywords:** broadcast, drop when seen, feedback, integer partition, online network coding, probability of innovativeness, randomized drop when seen

## Abstract

The *Drop when seen* (DWS) technique, an online network coding strategy is capable of making a broadcast transmission over erasure channels more robust. This throughput optimal strategy reduces the expected sender queue length. One major issue with the DWS technique is the high computational complexity. In this paper, we present a randomized version of the DWS technique (rDWS), where the unique strength of the DWS, which is the sender’s ability to drop a packet even before its decoding at receivers, is not compromised. Computational complexity of the algorithms is reduced with rDWS, but the encoding is not throughput optimal here. So, we perform a throughput efficiency analysis of it. Exact probabilistic analysis of *innovativeness* of a coefficient is found to be difficult. Hence, we carry out two individual analyses, *maximum entropy* analysis, *average understanding* analysis, and obtain a lower bound on the *innovativeness* probability of a coefficient. Based on these findings, *innovativeness* probability of a coded combination is analyzed. We evaluate the performance of our proposed scheme in terms of dropping and decoding statistics through simulation. Our analysis, supported by plots, reveals some interesting facts about *innovativeness* and shows that rDWS technique achieves near-optimal performance for a finite field of sufficient size.

## 1. Introduction

Reliable communication through packet erasure channels is one of the key challenges of modern wireless systems. Digital fountain codes [1,2,3] are a class of codes that can be used as a tool to ensure reliability in such scenario. Though originally network codes were invented for achieving multicast capacity of a multicast network which consists of some fixed capacity faultless links, it can also be used to deal with heterogeneous packet-erasure networks. Network coding is preferred over fountain codes in some cases as decoding is not needed in the intermediate nodes, and it is more composable across links over various network configurations.

The idea of network coding was proposed by Ahlswade et al. [4] in the context of network information flow where information is not just a commodity like water which is flowing through a pipeline network. Breaking the traditional convention of routing, incoming information packets to a node are coded and forwarded to the next nodes across outgoing links. The main goal of this code and forward technique is to increase the throughput of the network, which may seem surprising at first. To visualize this throughput enhancement, one can look at the one source two sink network presented in Figure 7 of Reference [4] and the corresponding discussion. A slightly different version of that network was later named as the butterfly network (see Figure 2 of Reference [5] for instance). With the help of the butterfly network, it was shown in [5] that the achievable network coding data rate is more than the routing data rate for multicast and multiple unicast flows. By extending the Max-flow Min-cut Theorem to network information flow, the work in [4] showed that optimum throughput of a multicast network can be achieved with network coding (which is not always possible with conventional routing). Reference [6] established that linear combination of incoming data packets at the intermediate nodes is sufficient to achieve multicast capacity, and this is termed linear network coding (LNC). A further illustration of this technique is random linear network coding (RLNC) [7] where the coding coefficients are chosen randomly from a finite field of sufficient size. Maximum achievable limit of capacity of a multicast network can also be attained with high probability with RLNC. Apart from the throughput and capacity benefit, network coding can improve latency, energy efficiency, network security, etc. in various networks and systems like lossy networks, ad-hoc sensor networks, delay tolerant networks, multicast networks, satellite communications, peer-to-peer content distribution systems, data storage systems, etc. [5,8,9,10,11,12,13,14].

A network is said to be solvable if each receiver in the network can recover their demands (set of packets, originated at the sources) from the received network coded packets [15]. When any link in a network carries a single data packet (raw or coded) and coding coefficients at each intermediate node are considered as vectors, it is termed as scalar network coding [16]. Similarly, when the links carry packet vectors instead of a single packet, and coding coefficients at intermediate nodes are considered as matrices, we call it vector network coding [16]. The latter has certain advantages over the previous linear network coding, such as higher ratio of total possible coding coefficients to total possible source messages [17]). Some of those advantages are summarized in [18]. The connection of field size with linear solvability of a network was first revealed in Reference [6], where it was shown that a multicast network is linearly solvable if the field size is sufficiently large. Since then, there has been extensive research on the effect of field size on scalar linear solvability and on the effect of field size and message dimension on vector linear solvability of various multicast and non-multicast network configurations [15,18,19,20,21,22,23,24,25]. Some recent work on this solvability issue has shown that not only the field size but also the order of the subgroups in the multiplicative group of the finite field are responsible for linear solvability of a multicast network [26]. For the rest part of this paper, by linear network code, we refer to scalar linear network code.

From the inception of RLNC, there are two basic modes of operation which are present in multicast or multiple unicast scenarios in a packet erasure wireless environment. For a large stream of data, it is quite a tough job to encode all packets of the stream because the computational complexity of encoding becomes pretty high. In a practical approach, data streams are divided into a fixed size of blocks and encoding is done within a block. These blocks are called generations [27], and the encoder moves to the next generation only when all packets of current generation are delivered to the receivers. This generation-based RLNC approach [28,29,30,31,32,33,34,35,36] has an inherent problem of large decoding delay because of the packet level coding. The second approach is slightly different from this and known as the systematic RLNC approach [37,38,39,40]. Here all packets of a generation are sent uncoded at first. After that, coded packets are sent to recover the packets which are lost due to channel erasure. The systematic approach is beneficial in a low loss network, but the performance becomes significantly poor with increase of erasure probability of channels, and the situation gets worse in terms of delivery delay when the receivers want in-order packet delivery. Several works are done in obtaining the decoding and delivery delay for both of the schemes from queueing as well as probabilistic point of view. Various works are also done to modify those approaches to overcome certain drawbacks or to make them fit to fulfill certain setup requirements or to pace up the transmission procedure [33,41,42].

While sending coded packets in the block RLNC or systematic RLNC approaches, the idea of *innovativeness* plays an important role. A coded packet is *innovative* to a receiver when the corresponding coefficient vector is not in the span of the coefficient vectors of previously received packets. When each receiver receives an *innovative* packet each time it receives a coded packet, we say the corresponding network coding strategy is throughput optimal. Those kinds of network coding strategies which produce *innovative* combinations in each attempt are said to have *innovation guarantee property* [43]. When we see a network where a coding strategy with *innovation guarantee property* is employed, data transmission in that network achieves optimal throughput and that network coding strategy is said to be a throughput optimal strategy. Li. et al. [6] showed that linear network codes are sufficient to achieve the optimal throughput in a multicast network. Now, if the network encoding strategy is not throughput optimal, the receivers need to collect some extra coded packets to fulfill their requirements even if the channels are erasure free. RLNC-based broadcast is an example of such a scenario where there is no issue of the network solvability. The broadcast shows near-optimal performance for a sufficiently large finite field [7]. A throughput optimal LNC scheme which deterministically chooses *innovative* coefficient for each packet in the linear combination by a polynomial time algorithm is known as deterministic LNC (DLNC) [44]. However, the DLNC algorithm is hard to implement without proper feedback from the receivers. One GF(2)-based throughput optimal LNC is proposed in [32] which can be equipped with block-based LNC. This is named triangular network coding, and it does not require any feedback from the receivers.

Effect of feedback can be exploited in LNC to increase the throughput optimality of encoding as well as to reduce the block level and packet level delay in various application-oriented scenarios. Some initial literature on this can be found in [45,46]. At the transmitter side, feedback from the receivers can be used to keep track of the receivers’ progression in terms of the acquired degrees of freedom (dof) [47]. In DLNC, these dofs are used in the online algorithm to form *innovative* packets at each time slot. Following this idea, References [31] and [47] proposed two ARQ-based throughput optimal encoding techniques. These techniques were equipped with fixed-generation-based situations [31,48] as well as with stochastic arrival-based situations [36,47,49,50]. Some adaptive and opportunistic algorithms were given in [51,52] where the reduction of decoding delay was given more priority rather than throughput optimality, and algorithms of these type form a new class of network code, termed as instantly decodable network code (IDNC). References [50,53,54] analyzed similar feedback-based schemes respectively in deadline-aware applications, in delay-tolerant applications, and for dynamic transmission rate adaptation for improved throughput-delay performance.

### 1.1. Motivation

Queueing analysis is one of the key tools for analyzing various performance measures of a network coded multicast or broadcast system. Numerous literature is available where queueing analysis is done over different kinds of network coded systems to find out improvements in various issues like throughput and delay benefit; trade-offs between coding gain and decoding delay; characterization of capacity region, stability region and throughput region; limitation in feedback’s usefulness etc. for various packet injection processes in sender queue (such as stochastic injection, constant data injection, and burst injection) [35,36,37,47,48,55,56,57]. Among those works, Reference [47] is the first to provide a technique that helps in keeping shorter expected sneder queue (SQ) lengths by exploiting the use of feedback.

In conventional generation-based multicast, the transmitter cannot drop any packet of a generation even if there is only one receiver left who has yet to gather some packets to decode the generation. This situation is exactly the same with throughput optimal and nonoptimal encodings. The transmitter drops the whole generation at a time when decoding has happened at each receiver. Therefore, this concept is termed the *drop when decoded* (DWD) scheme [43,47]. The main problem with the DWD technique is the high probability of blocking new incoming packets to the SQ. Practically, the SQ is of finite length, and the transmitter cannot accommodate an incoming packet in SQ if it is full. No new arrival to SQ can be taken place to the fully occupied queue until the current generation under transmission is dropped. In systematic encoding approach, the situation is a bit better in terms of dropping a fraction of a generation, but that is not guaranteed always. This is because different multicast channels may behave differently in the same time slot, and this heterogeneity arises even when channels are stochastically homogeneous. Employment of feedback in the above methods helps the transmitter keep track of each receiver’s degree of progression, but implementation of the scheme of dropping of packets from SQ before decoding at all receivers is not straightforward.

Sundararajan et al. came up with the notion of *seeing* a packet [43,47] and proposed the useful *drop when seen* (DWS) technique that, for the first time in the literature of network coding showed interest about dropping a packet before decoding the same at the receivers. They considered stochastic arrival at SQ and did a queueing theoretic analysis to show that, with their proposed scheme, the physical queue tracks the virtual queue [58,59] and the average queue length is minimized. The whole DWS technique is divided into two parts. One part does the queue management, and the other part does throughput optimal encoding. Theorem 8 and Theorem 11 of [43] establish the throughput optimality of the DWS technique and explain the conditions at which that optimality holds. The related discussion and results of [43] ensure that optimal throughput is obtained in a DWS-based single source, multiple sink multicasting, or broadcasting scenario.

Beside throughput optimality and efficient SQ management benefit, the issue of delivery and decoding delay is prominent in the DWS scheme. Analysis of the delay profile of this scheme was done extensively in [49,50]. Several works are done [50,53,60] to modify the DWS algorithm for delay control while keeping the main advantages of the technique. Reference [61] performed a statistical packet dropping analysis of a generation-based DWS broadcast system, and based on that analysis, provided a closer look at the delay profile for different decoding situations. A throughput-delay tradeoff is shown in [54] by careful and dynamic adaption of the transmission rate. Some other works adopted the idea of *seeing* in their proposed multicast models and showed the throughput and delay improvement of the models [34,35,55,57].

In DWS technique, the queue update module (QUM) at the sender performs efficient SQ management by keeping track of each receiver’s *knowledge space* [43,47] through perfect feedback. The QUM does the whole operation at the end of each slot so that the coding module (CM) can perform throughput optimal encoding on the queue content at the beginning of the next time slot. However, to keep track of each receiver’s *knowledge space*, QUM needs to do Gauss–Jordan elimination which is of high computational complexity. Thus the cost of efficient queue management of the DWS is paid in terms of high computational complexity. Moreover, the throughput optimal encoding algorithm which is executed at CM is just an instance of the polynomial time algorithm of DLNC encoding. Computational complexity of both the algorithms at QUM and CM is high and increases rapidly with the generation size, the number of the receivers and the size of the finite field under consideration in a generation-based DWS broadcast. This analysis is elaborately done in Section 3 of this paper. The above discussion motivates us to look for modified algorithms for QUM and CM which will be of less computational complexities, but the essence of the DWS technique will remain intact.

### 1.2. Contributions and Organization of the Paper

From the discussion of Section 1.1, one can infer that DWS algorithms are deterministic. In this paper, we propose two modified algorithms respectively for CM and QUM which are randomized. Hence, we name our modified technique as the *randomized drop when seen* (rDWS) technique. In a network coded multicast or broadcast, the receivers store the received packets at their buffer. When they gather sufficient packets, decoding happens, and decoded packets are transferred to intended applications. In other words, decoding occurs when a receiver’s *knowledge space* catches up with the *knowledge space* of the transmitter’s. Now, each receiver keeps track of its own *knowledge space*. In our proposed rDWS scheme, we exploit this fact and the advantage of feedback simultaneously. The transmitter needs not keep track of the *knowledge space* of the receivers. Instead, the dimension information of the spaces is enough to perform efficient SQ management as in DWS case. Here, the transmitter gets the dimension information through feedback (we consider NACK-based feedback) from the receivers.

We design our QUM algorithm based on the above discussion. This modified algorithm is computationally less expensive than the one in DWS because, here, the transmitter needs not perform Gauss–Jordan elimination *n* times (*n* is the number of broadcasting receivers) to keep track of each receiver’s *knowledge space*. Rather, the receivers compute their *knowledge space* at each slot and convey the dimension information of the spaces to the transmitter through feedback.

Now, for the coding module in rDWS case, throughput optimal encoding is not possible as the transmitter does not have the complete *knowledge space* information of the receivers. The QUM discards the packets which are *seen* by all receivers at the end of a slot. The CM does a random linear encoding with the next *unseen* packets (considering the next *unseen* packets of all receivers) at the beginning of the next slot. Therefore, our proposed algorithm is similar to conventional RLNC encoding [7]. Also, this randomized version of the CM algorithm is very simple and computationally less expensive than the one in the DWS case.

Employment of random linear encoding in our technique does not alter the network solvability issue [7]. However, we have to analyze the throughput efficiency of our randomized CM algorithm in terms of probability of getting an *innovative* combination, generated by the CM. We first analyze the probability of getting an *innovative* coefficient $\left(P_{C}\left(i\right)\right)$ for the $i^{th}$ *unseen* packet of SQ. *Innovative* coefficient of an *unseen* packet $p_{i}$ implies such a coefficient which is such that, when it is used in the linear combination by CM, dimension of each receiver’s (whose next *unseen* packet is $p_{i}$) *knowledge space* increases by one, upon successful reception of that combination. Mathematically, finding an exact expression of $P_{C}\left(i\right)$ is found to be difficult. Therefore, we do two different analyses of $P_{C}\left(i\right)$, namely, *maximum entropy* analysis and *average understanding* analysis, and provide a lower bound on $P_{C}\left(i\right)$. Based on this, we perform an analysis of the probability of getting an *innovative* combination (or *innovative* coded packet), formed by CM.

As the idea of dropping a packet from SQ even before decoding at the receivers is also present in our proposed scheme, we carry out a statistical performance analysis on packet dropping as well on decoding by means of simulation. We consider three metrics, the cumulative packet dropping probability, the average time to drop the last packet of a generation, and the cumulative decoding probability of a generation in order to inspect the performance of the rDWS scheme. In [7], we saw that the conventional RLNC shows near-optimal performance for a finite field of sufficient size. Similar conclusions can be drawn for the rDWS technique from our analytical and simulation results.

The rest of the paper is organized as follows: Section 2 describes the system settings and provides some preliminary definitions. In Section 3, detailed description of the existing DWS technique, the proposed rDWS technique, and their comparison in terms of computational complexity are given. Section 4 is concerned with how close the rDWS encoding is towards throughput optimality. In Section 4.1, analysis on the probability of getting an *innovative* coefficient for an *unseen* packet in the linear combination is presented. Using this, the probability of a linear combination being *innovative* is analyzed in Section 4.2. The statistical performance evaluation of the rDWS technique through simulation is done in Section 5. Finally, we conclude the paper in Section 6.

## 2. System Model

We consider a broadcasting communication scenario similar to [43]. A sender is trying to broadcast a series of data packets to *n* receivers over independent, discrete time, packet erasure wireless channels. A receiver can detect an erasure in the transmission. Packets arrive at the sender from some information source according to some stochastic process and are stored in the finite-lengthed sender queue. These stored packets are divided into fix-sized generations for encoding purposes. We assume that the SQ has at least one generation of packets before broadcasting starts. Instead of sending raw packets, the sender does linear network encoding (over a finite field of sufficient size) within a generation and transmits the coded packets. Only a single packet (raw or coded) can be sent over a time slot. For ease of explanation, we consider generation-based encoding. However our proposal and its analysis are equally applicable to non-generation-based encoding.

Each receiver has a finite length buffer to store the incoming packets. When a receiver collects a sufficient number of appropriate coded packets, decoding happens, and decoded packets are delivered to the intended applications.

In this paper, we are dealing with two network coding techniques, DWS and rDWS. To execute these online techniques, the sender needs to know each receiver’s state of progress at each time slot. Therefore, perfect error-free feedback is considered. In the DWS case, feedback is sent in the form of ACK from each receiver to convey successful reception (or no erasure in transmission). In the rDWS case, NACK is sent to indicate either erasure or reception of a *non-innovative* packet.

**Figure 1:**
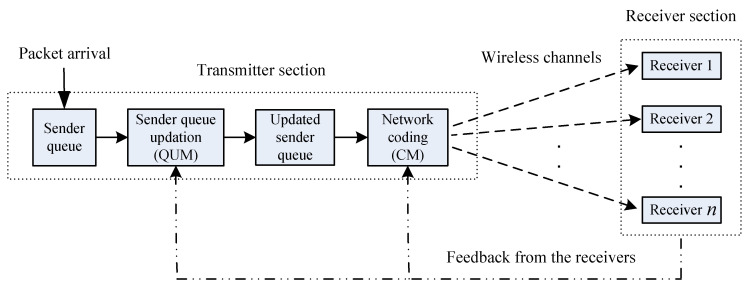
Block diagram of the components of the broadcasting transmission scenario.

In Figure 1, we outline our model for the broadcasting transmission scenario we will be studying in this paper. As mentioned in Section 1.1, the modules QUM and CM process the data packets at the transmitter side, which are then sent to the receivers through wireless channels. The roles of these two modules will be discussed in detail in Section 3.

**Figure 2:**
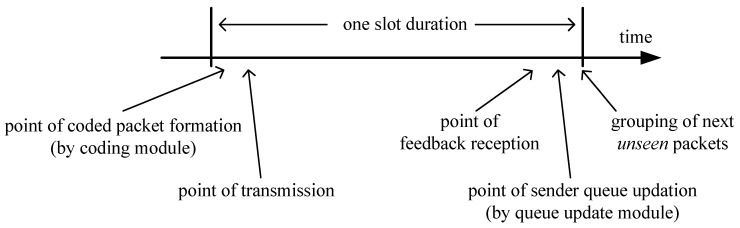
Relative timing of encoding, transmission, feedback reception, and sender queue updation points within a time slot.

Timing: We depict the timing occurrence of the events within a slot in Figure 2. The CM forms a linear combination using the next *unseen* packets of all the receivers at the beginning of a slot. The coded combination is sent just after that. We assume negligible propagation delay for simplicity. Also, the coded transmission reaches the receivers (if not erased at the channels) before they prepare and send feedback for that slot. Feedback from each receiver is received at the sender before the end of the same time slot. Hence, the feedbacks are guaranteed to convey current slot’s reception information. At the end of the slot, SQ content is updated by the QUM according to feedback, and then the next *unseen* packets to the receivers are grouped for the next slot’s encoding purpose.

Before going into the details of the techniques (DWS and rDWS), here we look at some preliminary definitions and necessary explanation.

### Definitions

- *Packet index*: Following in-order packet delivery (i.e., the packets are delivered in the same order they arrive at the SQ), the i^th^ packet which arrives at the SQ is said to have index *i*.
- *Coefficient vector of a coded packet*: The vector corresponding to a network coded packet or coded combination which consists of the coefficient of the raw packets which are involved in that combination.
- *Knowledge space of a node*: The vector space at a node which is the span of the coefficient vectors of the available packets at that node.
- *Innovative packet*: A network coded packet is *innovative* to a receiver if the corresponding coefficient vector does not belong to the receiver’s *knowledge space*. An *innovative* packet will always increase the dimension of a receiver’s *knowledge space* upon successful reception.
- *Hearing a packet*: A receiver has *heard* of a packet $p_{i}$ if it has received a linear combination involving $p_{i}$.
- *Seeing a packet*: A receiver has *seen* a packet $p_{i}$ if it can compute a linear combination of the form $p_{i}+\sum_{\forall k}\alpha_{k}p_{k}$ (k>i and $\alpha_{k}\in F_{q}$ for each *k*) based on the combinations it has received up to a slot.
- *Witness of a seen packet*: Linear combination of the form, $p_{i}+\sum_{\forall k}\alpha_{k}p_{k}$ (k>i and $\alpha_{k}\in F_{q}$ for each *k*), is the *witness* for receiver *j* of *seeing* $p_{i}$ which is denoted by $w_{j}\left(i\right)$.

**Example** **1.** *Suppose at a particular time slot SQ contains packets $p_{1},p_{2},p_{3}$ and $p_{4}$ and a receiver j has seen only $p_{1}$. For j, the next unseen packet is $p_{2}$. The CM makes a linear combination $l=\alpha_{1}p_{1}+\alpha_{2}p_{2}+\alpha_{3}p_{3}+\alpha_{4}p_{4}$, and broadcasts it along with the coefficient vector $\left(\alpha_{1},\alpha_{2},\alpha_{3},\alpha_{4}\right)$ in the header. On successful reception of l, j sends an ACK to the transmitter. Receiving the ACK, transmitter understands that j has seen $p_{2}$ and has only heard of $p_{3}$ and $p_{4}$, and its next unseen packet is $p_{3}$. The readers are encouraged to read [43] for more information.*

## 3. Existing DWS Technique and the Proposed rDWS Technique

As said before, our proposed technique is the randomized version of the conventional DWS scheme which is essentially a deterministic technique. Basic advantages of the DWS are two-fold. Firstly, it is throughput optimal, and secondly, it maintains a shorter expected SQ length throughout the transmission period. The essence of the DWS technique lies in the dropping of packets from SQ before decoding at receivers, and the QUM at the sender performs this operation. The other module, CM, does the throughput optimal encoding.

In the randomized version of the DWS method, we too have these two modules although their functions are modified. To understand the necessity of the rDWS technique and how we are incorporating randomization, we need to look at the DWS algorithms first. In Section 3.1, first we analyze the CM algorithm of the DWS technique. Then we provide the rDWS version of the algorithm in detail. A similar analysis of QUM algorithms (DWS and rDWS respectively) is given in Section 3.2.

The discussions of Section 3.1 and Section 3.2 compare the DWS algorithms with the rDWS counterparts which establish the usefulness of our proposed scheme over DWS technique.

### 3.1. The Coding Module

#### 3.1.1. DWS Case

In the DWS technique, the CM forms an *innovative* linear combination using the next *unseen* packets of the receivers. Algorithm 1 includes the work of CM in a step by step manner. Before the execution of the algorithm, CM groups the receivers with the same next *unseen* packets. Let R(i) denote the set of receivers of which the next *unseen* packet is $p_{i}$. In a generation-based encoding, each packet of the generation is the next *unseen* packet to at least one receiver in the worst-case situation. Therefore, we consider all possible R(i) where *i* varies from 1 to *m* (*m* is the generation size). Algorithm 1 deterministically chooses coefficients for each *unseen* packet (coefficient of $p_{i}$ in the combination is taken as $\gamma_{i}$) in such a way that it leads to an *innovative* combination, $l=\sum_{i=1}^{m}p_{i}\;\gamma_{i}$. The CM finds a coefficient $\alpha_{i}\;\left(\;\in F_{q}\right)$ to be *innovative* for $p_{i}$ if it is different from the coefficient of $p_{i}$ in $y_{j}$ for each j∈R(i). This ensures the increment of dimension of receiver *j*’s *knowledge space* upon successful reception of the coded packet.

**Algorithm 1** DWS Algorithm (The Coding Module)
1:Initialize $\gamma_{i}=0$, for i=1 to *m*2:**for**i=1 to *m* **do**3: **for** j=1 to |R(i)| **do**4:  Initialize $y_{j}=0$
5:  **for** k=1 to i−1 **do**6:   $y_{j}\leftarrow y_{j}+\alpha_{k}\;w_{j}\left(k\right)$
7:  **end for**
8: **end for**
9: **for** j=1 to *q* **do**10:  **for** k=1 to |R(i)| **do**11:   **if** coefficient of $p_{i}$ in $y_{k}=\alpha_{j}$ **then**12:    **Continue** loop for the next value of *j* at line 9 13:   **end if**
14:  **end for**
15:  $\gamma_{i}=\alpha_{j}$
16:  **Continue** loop for the next value of *i* at line 2 17: **end for**
18:
**end for**
19:Compute the linear combination, $l=\sum_{i=1}^{m}p_{i}\;\gamma_{i}$


Note that, if *j* is such a receiver that belongs to R(i) but has not *heard* of packet $p_{i}$, the corresponding $y_{j}$ does not have a $p_{i}$ term. Therefore any coefficient for $p_{i}$ is going to be *innovative* for *j*. Thus, the fate of the coefficient of $p_{i}$ in the final combination (*l*) depends on the receivers who have *heard* of $p_{i}$ in any of the previous slots. We will discuss more on this while analyzing the rDWS technique in Section 4.

Now, we look for the worst-case computational complexity of Algorithm 1. Maximum value of |R(i)| can be *n* which is the total number of receivers. At worst case, the loop starting at line 5 is executed m−1 times, and the algebraic expression of the witness of a specific packet in terms of that and other packets can contain at most *m* packets. Considering all these facts, overall complexity of Algorithm 1 is found to be $O(mn\left(m^{2}+q\right))$. Therefore, from complexity perspective, one can see that finding an *innovative* combination is very expensive if the number of receivers and generation size are high or even moderate.

#### Minimum Field Size Requirement

The minimum field size requirement for the throughput optimal DWS encoding is the same as the number of receivers (Theorem 8 of Reference [43]).

#### 3.1.2. rDWS Case

To reduce the computational complexity of Algorithm 1, we convert the previous deterministic algorithm into a randomized one. In conventional random linear network encoding [7] random coefficients are chosen from a finite field of sufficient size to form coded packets. This may not be optimal from the throughput efficiency perspective but is proven to be multicast capacity achieving. Here, the idea of RLNC is mixed with the original CM algorithm. The CM in the rDWS case picks random coefficients from a finite field for each next *unseen* packet of SQ and makes the linear combination (*l*). We present the CM algorithm of the rDWS technique in Algorithm 2 where the worst case scenario is considered as in the CM-DWS algorithm (which implies the involvement of all packets of a generation in the linear combination).

**Algorithm 2** rDWS Algorithm (The Coding Module)
1:Initialize $\gamma_{i}=0$, for i=1 to *m*
2:**for**i=1 to *m* **do**3: Pick an α randomly from $F_{q}$
4: $\gamma_{i}=\alpha$
5:
**end for**
6:Compute the linear combination, $l=\sum_{i=1}^{m}p_{i}\;\gamma_{i}$


We see the computational complexity of Algorithm 2 is O(m) which is independent of the total number of receivers or the field size. The reduction in computational complexity in CM algorithm from DWS to rDWS is obtained in the cost of employing *non-innovativeness* in encoding. Although it brings some extra delay in decoding, there is no issue from the network solvability aspect. Cruces et al. [30] formulated the probability of getting *Jinnovative* combinations out of *K* combinations (K≥J) as a function of field size for random DWD encoding. Similarly, we analyze our rDWS encoding in Section 4 to track the effect of randomization in throughput efficiency.

#### Minimum Field Size Requirement

As rDWS encoding is not throughput optimal, there is no minimum field size requirement actually. However, one can notice that if the field size is less than the number of receivers, there may be some situations where the probability of getting an *innovative* combination is zero (i.e., no proper choice is available in the pool of choices, depending on the finite field, that can make an *innovative* combination). Keeping this in mind, for our analysis, we only consider the extension fields of GF(2) of which the sizes are greater than or equal to the number of receivers. As an example, in Figure 6 (Section 4.2.1), the maximum number of receivers under consideration is 15. The nearest extension field of GF(2) of which the size is greater than or equal to 15 is GF($2^{4}$). Hence, we plot the graphs in Figure 6 with respect to field size, starting from $2^{4}=16$.

#### 3.1.3. Remarks

While describing the CM algorithms (Algorithms 1 and 2), we consider that all packets are involved in the linear combination *l*. This is not the case that always happens. At a particular slot, the loop starting at line 2 (Algorithms 1 and 2) is executed for those values of *i* which correspond to the index of the next *unseen* packets (for all receivers). In the worst case, *i* takes each value from 1 to *m* and the loop is executed *m* times. When all the receivers belong to a particular R(i) for some i∈{1,2,⋯,m}, the CM sends $p_{i}$ uncoded as there is no need to form a linear combination.

### 3.2. The Queue Update Module

#### 3.2.1. DWS Case

As said before, the QUM drops the packets which are *seen* by every receiver from the SQ and keeps only those packets of which is still *unseen* to some receiver(s). However, to maintain the SQ content with only *unseen* packets, the sender needs to keep track of each receiver’s *knowledge space*. A matrix of dimension m×m can hold one receiver’s *knowledge space* information for a generation size *m*. Also, the CM of the DWS technique needs the witness information of each receiver to perform encoding operation (line 6, Algorithm 1), and the witness information can be obtained from the rref (reduced row echelon form) of the *knowledge space* matrices.

Although the job of QUM is to manage sender queue, some portion of the algorithm (line 7, Algorithm 3) is executed with the help of the receivers. If a receiver successfully receives a coded packet, ACK is generated in constant time (i.e., with O(1) complexity) and is sent back to the transmitter. For now, we focus our attention only on the computational complexity of the algorithm and keep aside the propagation delay through wireless channels. Therefore, ACKs are generated in a parallel manner at all receivers. Based on the feedback, QUM updates *knowledge space* matrices ($B_{1},B_{2},\cdots ,B_{n}$). By finding the common *knowledge space*, the sender gets information about the packets which are *seen* at all receivers. Next, it drops the packets from SQ and updates *knowledge space* matrices.

The QUM performs its operations at each time slot. Also, upon successful reception of a coded packet, a receiver *sees* its next *unseen* packet. These two facts tell us that set *D* (line 15, Algorithm 3) contains only one packet. Hence, only a single packet can be dropped from SQ at a single slot.

One can note that line 1 of the algorithm is executed only once (before the start of broadcasting). After that, it is run from line 2 which is being continued until the broadcast of the current generation is finished. When ACK is received from receiver *j*, the row of $B_{j}$ corresponding to the next *unseen* packet of receiver *j* is updated with the coefficient vector of combination *l*, and there can be at most *m* components of that vector if all packets of the generation are involved in the creation of *l*. As the *knowledge space* matrices are of dimension m×m, there can be at most *m* packets in the set $D_{j}$ when all columns of its corresponding $B_{j}$ matrix are pivot columns. Apart from these facts, the arithmetic complexity of Gauss–Jordan elimination (which is $O(n^{3})$ for a n×n matrix) and the complexity of Algorithm 1 lead us to the worst case complexity of Algorithm 3 which is found as $O(mn\left(m^{2}+q\right))$.

There are basically two reasons behind the high computational complexity of Algorithm 3. The first one is to keep track of the receivers’ *knowledge space* and to perform Gauss–Jordan elimination. The second reason is the involvement of Algorithm 1 which is also computationally expensive. However, both of the facts are unavoidable as these are required to achieve the goals of the DWS technique. Now we present the QUM algorithm for rDWS scheme.

**Algorithm 3** DWS Algorithm (The Queue Update Module)
1:Initialize $B_{1},\;B_{2},\;\cdots ,\;B_{n}$ as m×m zero matrices 2:**if** SQ is empty **then**3: Do nothing4:
**else**
5: Call Coding Module (DWS), get linear combination *l* and transmit it 6:
**end if**
7:ACK generation at receivers 8:**for**j=1 to *n* **do**9: **if** ACK is received from receiver *j* **then**10:  Update the row of $B_{j}$ corresponding to the next *unseen* packet of receiver *j* with the coefficient vector corresponding to *l*
11:  Perform Gauss–Jordan elimination of $B_{j}$
12: **end if**
13: Find $D_{j}$ which is the set of packets corresponding to the pivot columns of $B_{j}$
14:
**end for**
15:Find $D=\cap_{j=1}^{n}\;D_{j}$
16:**for**j=1 to *n* **do**17: $B_{j}\leftarrow$ Change all elements of the pivot column and pivot row of $B_{j}$ corresponding to the packet in *D* to zero 18:
**end for**
19:Drop the packet in *D* from SQ


#### 3.2.2. rDWS Case

In rDWS, to form a random linear combination, the CM does not require the *knowledge space* information of the receivers. However, to maintain the SQ with only *unseen* packets, the *knowledge space* information is needed. More precisely, information about dimension of the *knowledge spaces* is sufficient for the QUM to do the job. To decode a generation, each receiver tracks its own *knowledge space* and stores that information at each slot. Now, the dimension information of the spaces can be conveyed to the transmitter through feedback. Hence, instead of matrices, we take *n* variables as counters in the QUM algorithm to store the dimension information.

Likewise in Algorithm 3, a receiver can store its *knowledge space* in a matrix. Following the CM algorithm of the rDWS case, a receiver’s *knowledge space* dimension will increase if the linear combination is *innovative* and the corresponding channel is not in erasure. A receiver sends a NACK to the sender if either it has received a *non-innovative* packet or the packet is erased in the channel. Here NACK is preferred instead of ACK (like in the DWS case) because, through NACK we can convey both the information, *non-innovativeness* and channel erasure. However, the NACK generation at receivers is not going to occur with constant complexity (O(1)). Algorithm 4 describes the NACK generation procedure at receiver *j*. Line 1 of the algorithm is executed only once (before the start of broadcasting). After that, it is run from line 2 which is being continued until the broadcast of the current generation is finished. Line 6 and line 9 ensure that, even in rDWS case, all packets are *seen* (and delivered) in the same order they arrive at SQ. The computational complexity of NACK generation (Algorithm 4) is found as $O(m^{3})$ because of the Gauss–Jordan elimination step at line 5.

**Algorithm 4** NACK generation at receiver *j*
1:Initialize $B_{j}$ as a m×m zero matrix 2:**if** linear combination *l* is received at the receiver **then**3: Find $D_{1}$ which is the set of packets corresponding to the pivot columns of $B_{j}$
4: Update the row of $B_{j}$ corresponding to the next *unseen* packet of receiver *j* with the coefficient vector corresponding to *l*
5: Perform Gauss–Jordan elimination 6: Find $D_{2}$ which is the set of packets corresponding to the *main diagonal* pivot columns (these are the pivot columns for which the pivot elements are in the main diagonal of a matrix) of updated $B_{j}$
7: **if** $D_{2}=D_{1}$ **then**
8:  Send NACK 9:  Make all elements of the last updated row of $B_{j}$ zero10: **end if**11:
**else**
12: Send NACK13:
**end if**



From the feedback of the receivers, the transmitter gets the dimension information and updates the counters ($k_{j}$ for j=1,2,⋯,n). From the counters’ value, the QUM identifies the packets which are *seen* by every receiver. Among these packets, the QUM drops the latest packet from SQ which was not dropped earlier. With the same argument given in the QUM-DWS case, one can observe that only a single packet can be dropped at a single slot from the SQ in rDWS case also.

The best part here is that the transmitter needs not keep track of the whole *knowledge space* of the receivers, but just the dimension. Also, NACK generation is a parallel process which can occur simultaneously at all receivers. This phenomenon reduces the overall computational complexity of the QUM algorithm of the rDWS which is presented in Algorithm 5.

**Algorithm 5** rDWS Algorithm (The Queue Update Module)
1:Initialize $k_{1},\;k_{2},\;\cdots \;,k_{n}$ as counters with value zero 2:**if** SQ is empty **then**3: Do nothing4:
**else**
5: Call Coding Module (rDWS), get linear combination *l* and transmit it6:
**end if**
7:NACK generation at receivers8:**for**j=1 to *n* **do**9: **if** NACK is received from receiver *j* **then**10:  Do nothing11: **else**12:  $k_{j}\leftarrow k_{j}+1$13: **end if**14:
**end for**
15:Find $k_{min}=\mathrm{minimum}\;\mathrm{element}\;\mathrm{of}\;\left\{k_{1},\;k_{2},\;\cdots \;,k_{n}\right\}$16:Drop the packet with index $k_{min}$ from SQ if it was not dropped at a previous slot


Next, we proceed for the computational complexity analysis of Algorithm 5. From line 2 to line 7, the computational complexity is $O(m^{3})$ (using the complexity of Algorithms 2 and 4). Complexity of line 16 is O(1). For the rest of the lines, the combined complexity is O(n) which is easy to deduce. Hence, the worst case complexity of Algorithm 5 is found as $O(m^{3}+n)$, which is less than the complexity of the QUM-DWS algorithm (Algorithm 3). This is a vast improvement.

While making this comparison between the DWS and rDWS techniques, the channel propagation delays of both the forward and feedback paths are kept aside. As the primary goal is to reduce the computational complexity while shifting from the DWS to rDWS method, some parameters like propagation delay are kept aside as they impart the same effect on the system in each case.

#### 3.2.3. Remarks

A broadcast system which uses rDWS method to send coded packets differs from a system with the DWS technique in work distribution between the transmitter and the receivers for proper flow of data in the system. In the DWS case, the transmitter is dependent on the receivers just for ACK reception. Apart from this, the transmitter does all the jobs of packet preparation and data transmission. Receivers only receive those packets according to the channel condition and wait for the proper time to decode the packets. Whereas, in the rDWS case, the receivers also play a significant role in packet processing at the transmitter. Here, the transmitter does not have exact information about receivers’ progress in terms of their *knowledge space*. The receivers keep themselves updated about their own *knowledge space* and convey the dimension information to the transmitter at the end of each slot. From the decoding point of view, this can be very useful if cooperation between receivers is allowed (though it is not always possible as the receivers may be situated very far from each other, geographically). Then they can help each other to speed up their progress in coded packet accumulation at very adverse channel (these are the channels through which they are connected with the transmitter) conditions.

An ACK-based QUM algorithm can also be constructed for the rDWS scheme. A receiver will send an ACK as feedback when it receives an *innovative* packet. One can infer that the computational complexity of an ACK-based QUM algorithm will be precisely the same as the computational complexity of Algorithm 5.

## 4. Throughput Efficiency Analysis of rDWS Technique

Because of the random selection of the coefficients, rDWS encoding is not throughput optimal. It was shown that, if we choose a random coefficient vector for a set of packets, the vector is going to be *innovative* with probability close to 1 with increasing field size [7]. In this section, our goal is to find out the probability with which a randomly picked-up coefficient vector is going to be *innovative* in rDWS scenario.

We pick up coefficients one by one for a linear combination in rDWS encoding. Each pick remains independent of others. Whether a coefficient is *innovative* depends on the receivers of which the next *unseen* packet is the one for which the coefficient is being chosen for (i.e., the *innovativeness* of the coefficient of $p_{i}$ depends on R(i)).

### 4.1. Probability of Innovativeness of a Picked-up Coefficient

Here, we analyze the probability of getting an *innovative* coefficient for a packet which will be included in the linear combination *l*. This analysis will lead us to find the probability of getting an *innovative* linear combination, which is equivalent to examining the throughput efficiency of the rDWS encoding.

If one looks at Algorithm 1, from line 5 to line 7, the CM finds the expression of $y_{j}$ for receiver j∈R(i). There can be two types of receivers in R(i). The receivers who have not *heard* of packet $p_{i}$ are of the first type, and the set of these receivers is denoted as T(i). The second type of receivers, who have *heard* of $p_{i}$ but have not *seen* it, belongs to set S(i). Therefore, R(i)=T(i)∪S(i). One can observe that R(1)=T(1) as S(1) is always a null set. Apart from the receivers which belong to some R(i) for i=1,2,⋯,m, there may exist some receivers which have *seen* all packets of the generation. The set of those receivers is denoted as E(m).

Now, the expression of $y_{j}$ for a receiver j∈T(i) does not contain a $p_{i}$ related term. Therefore, any choice of coefficient for $p_{i}$ in the linear combination is going to be *innovative* with respect to any receiver in T(i). Next, for a receiver j∈S(i), the corresponding $y_{j}$ have a $p_{i}$-related term and let the coefficient of $p_{i}$ in $y_{j}$ be $c_{j}\left(i\right)$. Let us also consider a vector c(i) that contains all the $c_{j}\left(i\right)$’s (for a fixed S(i) and j=1,2,⋯,|S(i)|). Therefore, $c\left(i\right)=(c_{1}\left(i\right),c_{2}\left(i\right),\cdots ,c_{\left|S(i)\right|}\left(i\right))$. All the $c_{j}\left(i\right)$’s in the vector c(i) may have the same values or may have different values from each other or any situation in between these two can occur. The total number of situations that can occur here is same as the integer partitions [62] of |S(i)|. When all coefficients are of the same value, total q−1 choices (excluding that value) will be a *good choice* for $\gamma_{i}$ (line 4, Algorithm 2). By *good choice*, we mean that the corresponding chosen coefficient is *innovative*. In the extreme case, when all $c_{j}\left(i\right)$’s are different, the total q−|S(i)| *good choices* are available. Therefore, the CM in rDWS case chooses an *innovative* coefficient with probability $\frac{1}{q-1}$ and $\frac{1}{q-|S(i)|}$ respectively in the terminal situations described above.

It is evident that, with rDWS technique, the probability of choosing an *innovative* coefficient for packet $p_{i}$ (which we denote by $P_{C}\left(i\right)$) only depends on S(i) as that probability is always one with respect to any receiver in T(i). For the same reason, $P_{C}\left(1\right)=1$ as |S(1)| is always zero. Now we illustrate our discussion on integer partitions of |S(i)| and the corresponding *good choices* available to the CM (rDWS) in the following example.

**Example** **2.** *Let us consider a broadcasting scenario where the packets are encoded with the rDWS technique. At the beginning of a particular time slot, it is found that |S(3)|=4 and the vector c(i) takes the form, $c\left(3\right)=(c_{1}\left(3\right),c_{2}\left(3\right),c_{3}\left(3\right),c_{4}\left(3\right))$. Depending on the channel erasures and the time slot under consideration, total five forms of c(i) can happen. These are $\left(\alpha_{1},\alpha_{2},\alpha_{3},\alpha_{4}\right)$, $\left(\alpha_{1},\alpha_{2},\alpha_{3},\alpha_{3}\right)$, $\left(\alpha_{1},\alpha_{1},\alpha_{2},\alpha_{2}\right)$, $\left(\alpha_{1},\alpha_{2},\alpha_{2},\alpha_{2}\right)$ and $\left(\alpha_{1},\alpha_{1},\alpha_{1},\alpha_{1}\right)$ where $\alpha_{1},\alpha_{2},\alpha_{3},\alpha_{4}$ are arbitrary and distinct elements of $F_{q}$. In the case of $\left(\alpha_{1},\alpha_{2},\alpha_{3},\alpha_{4}\right)$, all coefficients are different. Therefore, the total number of available good choices for $\gamma_{3}$ is q−4 and the probability of choosing an innovative coefficient for $p_{3}$ is $\frac{q-4}{q}$.*

*The form $\left(\alpha_{1},\alpha_{2},\alpha_{3},\alpha_{4}\right)$ corresponds to the integer partition |S(3)|=4=1+1+1+1, where the partition has a total of four different parts. Similarly the other forms of c(3) correspond to the partitions 4=1+1+2, 4=2+2, 4=1+3 and 4=4 respectively. For the partition 4=2+2, two receivers of S(3) have the same coefficient of $p_{3}$ (say $\alpha_{1}$) in their corresponding $y_{j}$’s. The rest of the two receivers also have the same coefficient (say $\alpha_{2}$), but it is different from the previous one (i.e., $\alpha_{1}\neq \alpha_{2})$. Therefore, each part of a partition denotes how many of the receivers in S(3) have the same coefficient of $p_{3}$ in their corresponding $y_{j}$ expressions. The total number of parts of a partition indicates the number of field elements which the CM must not pick as $\gamma_{3}$ in l. Thus, subtraction of the total number of parts of a partition from q gives total number of good choices for $\gamma_{3}$. Hence, the number of good choices available for both the partitions 4=2+2 and 4=1+3 is q−2. One can notice that, which receivers have the same coefficient of $p_{3}$ does not affect the number of good choices, whereas how many of the receivers have the same coefficient matters. In Table 1, a detailed description of all the partitions of |S(3)| is given.*

#### 4.1.1. Integer Partitions of |S(i)| and $P_{C}\left(i\right)$

So far, we have found out the probability of getting an *innovative* coefficient for $p_{i}$ corresponding to a particular integer partition of |S(i)|. Let us construct a set M(i) that contains all partitions of |S(i)| where partitions are arranged in decreasing order of number of parts. Also, each partition is structured in decreasing order of parts. If two partitions are of same number of parts, the partition with the larger first part is placed after the other one. If the first part is same for two partitions, the partition with the larger second part is placed later. This procedure is continued until the tie regarding the position of the partitions is broken. In this way, for |S(i)|=7, we get M(i)={(1+1+1+1+1+1+1),(2+1+1+1+1+1),(2+2+1+1+1),(3+1+1+1+1),(2+2+2+1),(3+2+1+1),(4+1+1+1),(3+2+2),(3+3+1),(4+2+1),(5+1+1),(4+3),(5+2),(6+1),(7)}. The total number of elements of M(i) is a function (ϕ) of |S(i)| which we write as, |M(i)|=ϕ(|S(i)|). We consider another function ψ(i,k) which gives number of parts of the $k^{th}$ element of M(i). One can see that ψ(i,1)=|S(i)| and ψ(i,|M(i)|)=1, for any *i*. Also, for a fixed *i*, the maximum value of ψ(i,k) is ${\left.\psi (i,k)\right|}_{\max}=\psi \left(i,1\right)=\left|S\left(i\right)\right|$, and minimum value of ψ(i,k) is ${\left.\psi (i,k)\right|}_{\min}=\psi \left(i,|M\left(i\right)|\right)=1$.

We consider a random variable $X_{i}$ which takes value *k* if the $k^{th}$ element of M(i) occurs. Let us define an event *I* which corresponds to getting an *innovative* coefficient for $p_{i}$.

(1) $$\begin{array}{c} \;P_{C}\left(i\right)=\mathrm{Pr}\left(I\right)=\sum_{k=1}^{\left|M(i)\right|}\mathrm{Pr}\left(I|X_{i}=k\right)\;\mathrm{Pr}\left(X_{i}=k\right)\\ \;\;\;\;\;\;\;\;\;\;\;\;\;\;\;\;\;\;\;\;\;\;\;\;\;\;\;\;\;\overset{\left(a\right)}{=}\sum_{k=1}^{\left|M(i)\right|}\left\{1-\frac{\psi (i,k)}{q}\right\}\mathrm{Pr}\left(X_{i}=k\right)\\ \;\;\;\;\;\;\;\;\;\;\;\;\;\;\;\;\;\;\;\;\;\;\;\;\;\;\;\;=\frac{1}{q}\sum_{k=1}^{\left|M(i)\right|}\left\{q-\psi (i,k)\right\}\;\mathrm{Pr}\left(X_{i}=k\right) \end{array}$$

where (a) follows from the inaugural discussion of Section 4.1. In Table 1, we have tabulated all the $\mathrm{Pr}(I|X_{i}=k)$’s for |S(i)|=4. Next, we look for the probability of occurrence of a partition of |S(i)| to get a complete expression of $P_{C}\left(i\right)$ according to (1).

#### Probability of Occurrence of a Partition of |S(i)|

The probability $\mathrm{Pr}(X_{i}=k)$ depends on certain issues like broadcast settings, channel erasure probabilities, the time gap between the starting time slot of broadcasting and the slot under consideration. By broadcast settings, we mean the number of receivers, the size of the generation etc. These factors have an influence on $c_{j}\left(i\right)$ heterogeneity (the dissimilarity between the $c_{j}\left(i\right)$’s where j=1,2,⋯,|S(i)|), which has direct impact on $\mathrm{Pr}(X_{i}=k)$.

If the erasure probabilities are very low and close to zero, the absolute $c_{j}\left(i\right)$ heterogeneity is very low. All the receivers are close to each other in terms of progress in getting coded packets, and the corresponding witness functions ($w_{j}\left(i\right)$ for j=1,2,⋯,|S(i)|) are also similar. Therefore, we can say that a partition of |S(i)| with more number of parts is less likely to occur than a partition with a fewer number of parts. In contrast, when erasure probabilities are high, $c_{j}\left(i\right)$ heterogeneity is also high. By similar logic, here a partition with more number of parts is more likely to occur than a partition with a fewer number of parts. These two are extreme situations that can happen. Any other situation may happen depending on the erasure probabilities and the situation lies in between the extreme cases. Hence, no perfect ordering between the $\mathrm{Pr}(X_{i}=k)$’s for k=1,2,⋯,|M(i)| exists.

If a considered time slot is beyond a particular value from the beginning of broadcasting, the $c_{j}\left(i\right)$ heterogeneity becomes prominent even for low channel erasure probabilities.

Now, for a suitable broadcast setting and moderate erasure probabilities, at a sufficiently large time slot, we focus our attention on the set S(i). If we consider a partition of |S(i)| as a state of the receivers in S(i), the path through which the receivers have reached that state is a |S(i)|-dimensional random walk which is mathematically difficult to track [49,50]. Therefore, finding an exact expression of $\mathrm{Pr}(X_{i}=k)$ is clearly a daunting task. However, we perform three different analysis of $\mathrm{Pr}(X_{i}=k)$ to get some insight into the statistical understanding of $P_{C}\left(i\right)$ according to (1).

#### 4.1.2. Maximum Entropy of $X_{i}$ and $P_{C}\left(i\right)$

Each value the random variable $X_{i}$ takes, corresponds to a partition of |S(i)|. From the previous discussion, it is clear that mathematically tracking the uncertainty of partitions is a tough job. Nevertheless, we can find the maximum uncertainty of the partitions. From the information theoretic view, maximum uncertainty of the partitions is same as the maximum entropy of the discrete random variable $X_{i}$ (i.e., $H(X_{i}))$. It is well-known that $H(X_{i})$ is maximum when $X_{i}$ follows uniform distribution [63]. Thus, we get $\mathrm{Pr}\left(X_{i}=k\right)=\frac{1}{\left|M(i)\right|}$. Applying this in (1), we get the probability of choosing an *innovative* coefficient for packet $p_{i}$ for maximum uncertainty of partitions as follows:

(2) $$\begin{array}{c} P_{CM}\left(i\right)=\frac{1}{q|M(i)|}\sum_{k=1}^{\left|M(i)\right|}\left\{q-\psi (i,k)\right\} \end{array}$$

**Figure 3:**
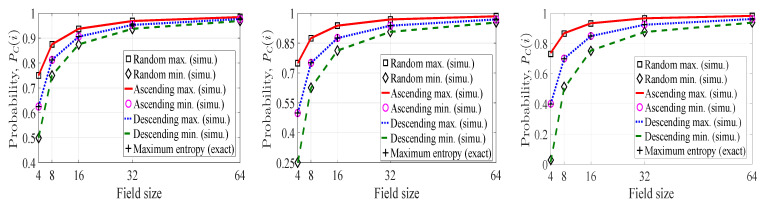
Probability of *innovativeness* of a picked-up coefficient, $P_{C}\left(i\right)$, versus field size where |S(i)|=2 (left subfigure), |S(i)|=3 (middle subfigure) and |S(i)|=4 (right subfigure).

In Figure 3, we have plotted the probability of *innovativeness* of a picked-up coefficient for packet $p_{i}$ ($P_{C}\left(i\right)$) with respect to field size for four different considerations of the probability $\mathrm{Pr}(X_{i}=k)$ (for a fixed S(i) and k=1,2,⋯,|M(i)|). The first consideration corresponds to the case where no perfect ordering of the $\mathrm{Pr}(X_{i}=k)$’s exists, and we generate the $\mathrm{Pr}(X_{i}=k)$’s for each *k* according to some arbitrary distribution in a *Monte Carlo* simulation environment. We plot the maximum and minimum values of $P_{C}\left(i\right)$ among $10^{7}$ runs according to (1) and denote these two values as *Random max.*, and *Random min.* in the figures. In the second case, we consider high $c_{j}\left(i\right)$ heterogeneity. This time, the $\mathrm{Pr}(X_{i}=k)$’s are also generated according to some arbitrary distribution with the consideration that a partition with more number of parts is more likely to occur than a partition with a fewer number of parts. To avoid ambiguity in choosing probability of two partitions of same number of parts, we consider $\mathrm{Pr}\left(X_{i}=k\right)>\mathrm{Pr}\left(X_{i}=k+1\right)$ as the partitions are contained in M(i) in such a way that, for any two consecutive partitions, the partition corresponding to more $c_{j}\left(i\right)$ heterogeneity is placed before the partition with less $c_{j}\left(i\right)$ heterogeneity. Again, we plot the maximum and minimum values of $P_{C}\left(i\right)$ among $10^{7}$ runs according to (1) and denote these two values as *Descending max.* and *Descending min.* in the figures. Similarly, *Ascending max.* and *Ascending min.* points in the plots denote the maximum and minimum values of $P_{C}\left(i\right)$ for low $c_{j}\left(i\right)$ heterogeneity consideration. Lastly, $P_{CM}\left(i\right)$ is plotted according to (2).

There are three subfigures in Figure 3 where we have depicted the probabilities with respect to field size. From left to right, the subfigures correspond to |S(i)|=2, |S(i)|=3 and |S(i)|=4 respectively. In each case, the probabilities increase as we increase the field size. Therefore, the chance of getting an *innovative* coefficient for $p_{i}$ can be made arbitrarily close to one for a field of sufficient size. When we move from the left to the right subfigure, the $c_{j}\left(i\right)$ heterogeneity gets increased. As a result, the $P_{C}\left(i\right)$’s decrease from left to right for a particular field size. For instance, $P_{C}\left(i\right)$ values corresponding to *Descending max.* curve at field size 16 are 0.91, 0.88 and 0.85 (approximate values) respectively from the left to the right subfigure.

From Figure 3, we observe one more interesting fact. The overall range of the $P_{C}\left(i\right)$ values for *Random case* is split by the *Maximum entropy* curve in each subfigure. The upper range corresponds to the $P_{C}\left(i\right)$ values of *Ascending case*, whereas the lower range corresponds to the $P_{C}\left(i\right)$ values of *Descending case*. This occurrence is because of the fact that maximum entropy corresponds to the uniform distribution which implies equal probability for each partition of |S(i)|. Thus, *Maximum entropy* case acts as the boundary between *Ascending* and *Descending* cases.

#### 4.1.3. Average Understanding of *Innovativeness* and $P_{C}\left(i\right)$

We have seen the likelihood of a partition depends on the erasure probabilities. Maximum entropy case implies maximum uncertainty of partitions, and this happens when all partitions are equally probable. If we consider the partition which is denoted by $X_{i}=\left|M\left(i\right)\right|$, only a single part is available. For a finite field of size *q*, the number of c(i) vectors that correspond to partition $X_{i}=\left|M\left(i\right)\right|$ is *q*. As a whole, total $q^{\left|S(i)\right|}$ vectors can result for a particular |S(i)|. To have an overall understanding of the receivers of S(i), we should look at all these $q^{\left|S(i)\right|}$ vectors. As discussed previously, no perfect ordering between the $\mathrm{Pr}(X_{i}=k)$’s exists. Here, we assume a broadcast set up and a time instance for which all $q^{\left|S(i)\right|}$ vectors are equally probable. The analysis of the probability of *innovativeness* for such an assumption gives an average understanding of the system irrespective of the physical relevance of the assumption.

We take a new function ξ(i,k) which gives the total number of c(i) vectors corresponding to the partition $X_{i}=k$. Using ξ(i,k), we rewrite (1) to get the probability of *innovativeness* for uniform distribution of the c(i) vectors as:

(3) $$\begin{array}{c} \;P_{CU}\left(i\right)=\frac{1}{q}\sum_{k=1}^{\left|M(i)\right|}\left\{q-\psi (i,k)\right\}\xi \left(i,k\right)\;\;\frac{1}{q^{\left|S(i)\right|}}\\ \;\;\;\;\;\;\;\;\;\;\;\;\;\;\;\;\;=\frac{1}{q^{|S(i)|+1}}\sum_{k=1}^{\left|M(i)\right|}\left\{q-\psi (i,k)\right\}\xi \left(i,k\right) \end{array}$$

**Lemma** **1.** *Let us consider a partition of |S(i)| as Z: = $\sum_{v=1}^{V}a_{v}b_{v}$, where part $b_{v}$ is present $a_{v}$ times and $b_{v}>b_{v+1}$ (for ∀v∈{1,2,⋯,V−1}). If the finite field under consideration is $F_{q}$, the total number of possible c(i) vectors corresponding to Z is given in (4), where $A=\sum_{v=1}^{V}a_{v}$, and ${}^{q}P_{A}$ represents A permutations of q.*

(4) NZ=1a1!a2!⋯aV!|S(i)|b1|S(i)|−b1b1⋯|S(i)|−(a1−1)b1b1·|S(i)|−a1b1b2|S(i)|−a1b1−b2b2⋯|S(i)|−a1b1−(a2−1)b2b2⋯|S(i)|−a1b1−⋯−aV−1bV−1bV⋯|S(i)|−a1b1−⋯−(aV−1)bVbV·qPA

**Proof.**  For partition Z, part $b_{v}$ is present $a_{v}$ times. Therefore for each *v*, there are $a_{v}$ groups where each group consists of $b_{v}$ receivers. The first group of $b_{1}$ receivers can be chosen from |S(i)| receivers in |S(i)|b1 many ways. Considering the second group of $b_{1}$ receivers, they can be chosen in |S(i)|−b1b1 many ways. Proceeding in this way, the last group of $b_{1}$ receivers can be chosen in |S(i)|−(a1−1)b1b1 many ways. All of these $a_{1}$ choices are independent because the broadcasting channels and the receivers are independent. Now, these $a_{1}$ choices can be permuted among themselves in $a_{1}!$ ways. Therefore, the total number of available choices for first $a_{1}b_{1}$ receivers is |S(i)|b1|S(i)|−b1b1⋯|S(i)|−(a1−1)b1b11a1!. Similar arguments can be shown for the next $a_{2}b_{2},a_{3}b_{3},\ldots ,a_{V}b_{V}$ receivers.

Now, a group hall receivers withinave the same coefficient, $c_{j}\left(i\right)$, in their corresponding $y_{j}$ equation. The total number of groups corresponding to partition Z is $A=\sum_{v=1}^{V}a_{v}$. Therefore, the total number of different coefficients corresponding to Z is also *A*. These coefficients are elements of finite field $F_{q}$. In a pool of *q* field elements, *A* elements can be permuted in ${}^{q}P_{A}$ ways. Thus, we get the total number of possible c(i) vectors corresponding to Z as in (4). □

**Example** **3.** *Let us consider a partition of |S(i)|=10 as, $\left|S\left(i\right)\right|=10=\sum_{v=1}^{V=3}a_{v}b_{v}$, where $b_{1}=3,b_{2}=2,b_{3}=1$, and $a_{1}=1,a_{2}=2,a_{3}=3$. If the size of the finite field under consideration is q=11, from (4), we get the total number of c(i) vectors corresponding to the partition given above as, 4,191,264,000.*

Lemma 1 provides the total number of possible c(i) vectors corresponding to partition Z. As Z is a partition of |S(i)|, Z corresponds to a partition $X_{i}=k$ for some k∈{1,2,⋯,|M(i)|}. Therefore, we can find out ξ(i,k) for each *k* using Lemma 1. Next, from (3), we get the probability $P_{CU}\left(i\right)$.

#### 4.1.4. Lower Bound on $P_{C}\left(i\right)$

In this section, we provide a lower bound on the probability $P_{C}\left(i\right)$. From (1) we get,

(5) $$\begin{array}{cc} \;P_{C}\left(i\right) & \overset{\left(a\right)}{\geq }\frac{1}{q}\sum_{k=1}^{\left|M(i)\right|}\left[q-\max_{\forall k}\left\{\psi (i,k)\right\}\right]\;\mathrm{Pr}\left(X_{i}=k\right)\\ \;\; & \overset{\left(b\right)}{=}\frac{1}{q}\sum_{k=1}^{\left|M(i)\right|}\left\{q-\psi (i,1)\right\}\;\mathrm{Pr}\left(X_{i}=k\right)\\ \;\; & \overset{\left(c\right)}{=}\frac{1}{q}\sum_{k=1}^{\left|M(i)\right|}\left\{q-|S(i)|\right\}\;\mathrm{Pr}\left(X_{i}=k\right)\\ \;\; & \overset{\left(d\right)}{=}\frac{\left\{q-|S(i)|\right\}}{q}\sum_{k=1}^{\left|M(i)\right|}\mathrm{Pr}\left(X_{i}=k\right)\\ \; & \overset{\left(e\right)}{=}\frac{q-|S(i)|}{q} \end{array}$$

where (a) is straightforward, (b) and (c) follow from the discussion of Section 4.1.1, (d) is true because the taken out term is constant over *k*, and (e) is correct because one of the partitions must occur. Equation (5) gives a lower bound on $P_{C}\left(i\right)$ which we write as:

(6) $$\begin{array}{c} P_{CL}\left(i\right)=\frac{q-|S(i)|}{q} \end{array}$$

Intuitively, one can understand that the probability of *innovativeness* is minimum when the partition $X_{i}=1$ results. At this situation, the number of *good choices* left is minimum. The lower bound is important because it helps to find the maximum time required to *see* a packet for a receiver. As decoding means *seeing* all packets of a generation [61], we can find maximum time to decode a generation for a receiver as well as for the whole receiver system. In short, $P_{CL}\left(i\right)$ helps in characterizing the decoding delay [49,50,53] profile of the broadcasting system.

**Figure 4:**
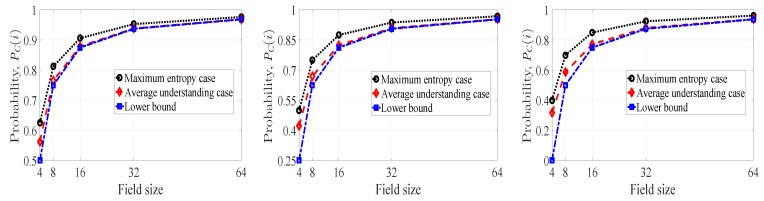
Probability of *innovativeness* of a picked-up coefficient $\left(P_{CM}\left(i\right),\;P_{CU}\left(i\right)\;\mathrm{and}\;P_{CL}\left(i\right)\right)$ versus field size where |S(i)|=2 (left subfigure), |S(i)|=3 (middle subfigure) and |S(i)|=4 (right subfigure).

In Figure 4, we have plotted the probability of *innovativeness* of a picked-up coefficient for packet $p_{i}$ with respect to field size for the maximum entropy case ($P_{CM}\left(i\right)$), the average understanding case ($P_{CU}\left(i\right)$), and the lower bound ($P_{CL}\left(i\right)$). There are three subfigures where we have depicted the probabilities with respect to field size. From left to right, the subfigures correspond to |S(i)|=2, |S(i)|=3 and |S(i)|=4 respectively. In each case, the probabilities increase as we increase the field size. Therefore, the chance of getting an *innovative* coefficient for $p_{i}$ can be made arbitrarily close to one for a field of sufficiently large size. When we move from the left to the right subfigure, the $c_{j}\left(i\right)$ heterogeneity increases. As a result, the $P_{C}\left(i\right)$ value decreases from left to right for fixed field size. For instance, the $P_{CU}\left(i\right)$ values corresponding to average understanding curve at field size 16 are 0.88, 0.82, 0.77 (approximate values) respectively from the left to the right subfigure.

One can observe that, the $P_{CM}\left(i\right)$ value is greater than the $P_{CU}\left(i\right)$ value in each subfigure for fixed field size. Therefore, the maximum uncertainty of the partitions of |S(i)| leads to better probabilistic performance than the average case scenario in terms of *innovativeness* of the chosen coefficient.

### 4.2. Probability of Innovativeness of a Linear Combination

So far we have analyzed the probability of *innovativeness* of a coefficient corresponding to a packet which is a part of the final linear combination. Based on that analysis, here our goal is to find out the probability of *innovativeness* of the linear combination to get an insight on the throughput performance of the rDWS encoding.

Let us consider the CM is making a combination *l* with only two *unseen* packets, $p_{i_{1}}$ and $p_{i_{2}}$, at a particular time slot. The probability of *innovativeness* of the combination *l* ($P_{L}$) is same as the joint probability of *innovativeness* of the coefficients of $p_{i_{1}}$ and $p_{i_{2}}$ $\left(\mathrm{the}\;\mathrm{coefficients}\;\mathrm{are}\;\gamma_{i_{1}}\;\mathrm{and}\;\gamma_{i_{2}}\;\mathrm{respectively}\right)$ which we denote as $P_{C}\left(i_{1},i_{2}\right)$. Now, the *innovativeness* probability of $\gamma_{i_{1}}$ depends on the receivers of $R(i_{1})$, whereas the *innovativeness* probability of $\gamma_{i_{2}}$ depends on the receivers of $R(i_{2})$. For any two receivers $j_{1}$ and $j_{2}$ (where $j_{1}\in R\left(i_{1}\right)$ and $j_{2}\in R\left(i_{2}\right))$, the channels through which they are connected to the transmitter are independent. Also, no co-operation is allowed between $j_{1}$ and $j_{2}$. Therefore we infer that, $P_{C}\left(i_{1},i_{2}\right)=P_{C}\left(i_{1}\right)\cdot P_{C}\left(i_{2}\right)$.

In the worst-case situation, the final combination *l* contains all packets of the generation. Though the worst-case situation may not be very likely to happen, to compare rDWS with DWS from a generation-based broadcasting perspective, it is considered that *l* consists of all packets. Now, extending the previous argument for two packets ($p_{i_{1}},p_{i_{2}}$) to all *m* packets of the generation, we obtain the probability of *innovativeness* of a linear combination as follows:

(7) $$P_{L}=P_{C}\left(1,2,\cdots ,m\right)=\prod_{i=1}^{m}P_{C}\left(i\right)\overset{\left(a\right)}{=}\;\frac{1}{q^{m-1}}\prod_{i=2}^{m}\;\sum_{k=1}^{\left|M(i)\right|}\left\{q-\psi (i,k)\right\}\;\mathrm{Pr}\left(X_{i}=k\right)$$

where (a) follows from (1) and $P_{C}\left(1\right)=1$. DWS is a throughput optimal technique where each linear combination made by CM is *innovative*. The $P_{L}$ helps in analyzing how close the rDWS encoding is towards the optimal DWS encoding.

#### 4.2.1. Special Cases

In Section 4.1, we carried out three individual analyses of $P_{C}\left(i\right)$. If we put the expression of $P_{CM}\left(i\right)$ in place of $P_{C}\left(i\right)$ in (7), we get the probability of *innovativeness* of *l* corresponding to Section 4.1.2, and we denote the probability as $P_{LM}$. Similarly, we obtain the expression of $P_{L}$ with respect to Section 4.1.3 and Section 4.1.4 which we respectively denote as $P_{LU}$ and $P_{LL}$. Among these probabilities, here, we emphasize $P_{LL}$ as this particular probability provides a deeper understanding of the least efficient throughput performance of the rDWS encoding. From (6) and (7), we get the expression of $P_{LL}$ as follows:

(8) $$\begin{array}{c} P_{LL}=\frac{1}{q^{m-1}}\prod_{i=2}^{m}\left\{q-|S\left(i\right)|\right\} \end{array}$$

#### Analysis of $P_{LL}$ and the Minimum Value

So, it is evident that $P_{LL}$ provides a lower bound on the probability of getting an *innovative* linear combination. It can be noted from (8) that $P_{LL}$ depends on the field size and on the |S(i)|’s for i=2,3,⋯,m. If one can find the minimum value of $P_{LL}$, it will lead us to the worst-case behavior of the rDWS technique in terms of throughput efficiency. Therefore, in this section, we aim to find out the minimum value of $P_{LL}$. The probability of *innovativeness*, $P_{C}\left(i\right)$, is always one with respect to a receiver who is *unheard* of its next *unseen* packet $p_{i}$. While searching for the minimum value of $P_{LL}$, we consider that each |S(i)| is a nonzero integer (except for |S(1)| which is always zero). This is to make sure that the probability of choosing an *innovative* coefficient for none of the packets of the generation is one, and each $P_{C}\left(i\right)$ (except for $P_{C}\left(1\right)$ which is always one) has some contribution in $P_{LL}$. Also, none of the |S(i)|’s (i=2,3,⋯,m) can be more than n−m+2 as the number of receivers is *n*.

For fixed values of number of receivers *n*, generation size *m*, and field size *q*, finding the minimum value of $P_{LL}$ is a standard optimization problem of the following form:

(9) $$\begin{array}{c} \mathrm{Minimize}:\;\frac{1}{q^{m-1}}\prod_{i=2}^{m}\left\{q-|S\left(i\right)|\right\}\\ \mathrm{Subject}\;\mathrm{to}:\;\sum_{i=2}^{m}\left|S\left(i\right)\right|\;\leq n \end{array}$$

It is a nonlinear integer programming which we reframe as the following:

$$\begin{array}{c} \min\;f\left(x\right)=\prod_{i=1}^{M}\left(q-x_{i}\right)\;\;\;s.t.\;\sum_{i=1}^{M}x_{i}\;\leq N\;, \end{array}$$

where,

(10) $$M=m-1,N=n,x=\left(x_{1},x_{2},\cdots ,x_{M}\right),x_{i}=\left|S\left(i+1\right)\right|,x_{i}\in \left\{1,2,\cdots ,N-M+1\right\}\;\;\mathrm{for}\;i\in \left\{1,2,\cdots ,M\right\}$$

To solve the integer program, first we look into the following consideration. The objective function f(x) has the same value for two vectors $x_{1}$ and $x_{2}$ if they are just element-wise permutation of one another. We call $x_{1}$ and $x_{2}$ as *equivalent* vectors. For instance, (3,1,2), (3,2,1), (1,3,2), (2,3,1), (2,1,3) and (1,2,3) form a group of *equivalent* vectors in the three-dimensional space. In a group of *equivalent* vectors, the vector whose elements are ordered in a non-increasing order from the first to the last element is denoted as the *representative* of that group. Thus, (3,2,1) is the *representative* of the previously mentioned *equivalent* vectors.

**Figure 5:**
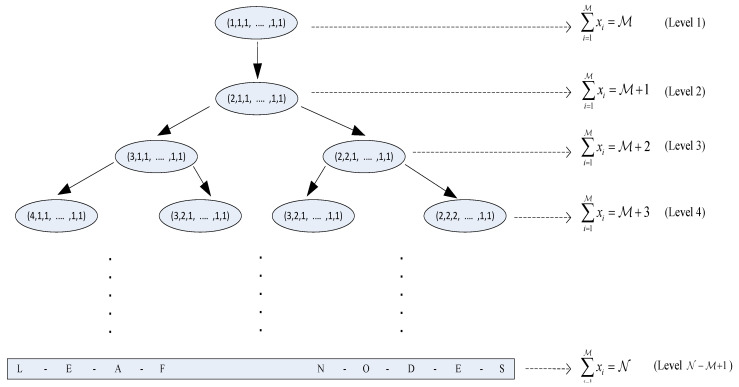
Tree corresponding to Section 4.2.1 where *representative* vectors represent nodes. The constraint corresponding to each level of the tree is written at the right.

The nonlinear programming problem is solved with a method which is quite similar to the famous *Branch and Bound* method [64]. A tree is constructed with only the *representatives* of each possible *equivalent* vector group which is depicted in Figure 5 for M-dimensional case. Starting node of the tree is x=(1,1,⋯,1) because $x_{i}\in \left\{1,2,\cdots ,N-M+1\right\}$ for each *i* and the lower bound on the constraint is $\sum_{i=1}^{M}x_{i}\;=M$. Second level of the tree corresponds to constraint $\sum_{i=1}^{M}x_{i}\;=M+1$, and only one node corresponding to vector (2,1,1,⋯,1) is possible. Therefore, the node (2,1,⋯,1) is the only child of (1,1,⋯,1). A node, $x=(x_{1},x_{2},\cdots ,x_{M})$, at level *h* (which corresponds to the constraint $\sum_{i=1}^{M}x_{i}\;=M+h-1$) can have at most M children at level h+1 which are $\left(x_{1}+1,x_{2},\cdots ,x_{M}\right)$, $\left(x_{1},x_{2}+1,\cdots ,x_{M}\right),\cdots ,\left(x_{1},x_{2},\cdots ,x_{M}+1\right)$. But, the number of children is much less because we only keep the *representative* vectors.

Now, we look at the positioning of the children of a particular parent node. The child whose first component of its vector is the largest among all is going to be the leftmost child. If the first component is same for two or more children, the child with the second largest component will be the leftmost child. This procedure continues until the leftmost child is decided. Once the leftmost position is decided, we proceed for the second leftmost position and the same rule is followed. We iteratively apply the rule to get the position of all children of a parent at each level and eventually obtain the complete tree structure as in Figure 5.

The leaf nodes of the tree correspond to the constraint $\sum_{i=1}^{M}x_{i}\;=N$. Now, our goal is to find the node or the nodes for which the cost of the objective function f(x) is minimum.

**Lemma** **2.** *Cost of f(x) associated with a node at level h is greater than the cost of f(x) corresponding to any of its children.*

**Proof.**  Let the cost of f(x) of a node at level *h* is $W_{h}$. We arbitrarily choose one of its children (which are at level h+1), and consider the associated cost as $W_{h+1}$. The relation between $W_{h}$ and $W_{h+1}$ is,

(11) $$\begin{array}{c} W_{h+1}=\frac{q-x-1}{q-x}\;W_{h} \end{array}$$

Depending on the position of the parent node at level *h* and the position of the considered child at level h+1, the value of *x* is from the set {1,2,⋯,N−M}. As the field size is greater than or equal to the number of receivers (Minimum field size requirement, Section 3.1.2), we get q≥N. Also, M≥2 because generation size must be greater than or equal to two. Now from (11), we get $W_{h+1}<W_{h}$. □

**Lemma** **3.** *The node at level h corresponding to vector (h,1,⋯,1) is the leftmost node at that level, and it has a child which is associated with vector (h+1,1,⋯,1) at level h+1.*

**Proof.**  The proof is trivial, and we omit it. □

**Proposition** **1.** *Starting from level 1 of the tree in Figure 5, we obtain the following order:*

(12) $$\begin{array}{c} f\left(x\right){|}_{x=(1,1,\cdots ,1)}>f\left(x\right){|}_{x=(2,1,\cdots ,1)}>f\left(x\right){|}_{x=(3,1,\cdots ,1)}>\cdots >f\left(x\right){|}_{x=(N-M+1,1,\cdots ,1)} \end{array}$$

**Proof.**  This directly follows from Lemma 2 and 3. □

**Proposition** **2.** *The leftmost node at level h corresponds to the minimum cost of the objective function f(x) at that level.*

**Proof.**  We prove this by induction on level *h*.

Base case: For level 1 and 2, single nodes are present. So, the proposition is true by default for level 1 and 2. In level 3, there are two nodes which are respectively associated with vectors (3,1,1,⋯,1) and (2,2,1,⋯,1). Cost function associated with the first vector is $f\left(x\right){|}_{x=(3,1,\cdots ,1)}=\left(q-3\right)\left(q\;-\;1\right){\left(q\;-\;1\right)}^{M-2}=\left(q^{2}-4q+3\right){\left(q-1\right)}^{M-2}$ whereas, the same for the second vector is $f\left(x\right){|}_{x=(2,2,\cdots ,1)}=\left(q-2\right)\left(q-2\right){\left(q-1\right)}^{M-2}=\left(q^{2}-4q+4\right){\left(q-1\right)}^{M-2}$. Clearly, $f\left(x\right){|}_{x=(3,1,1,\cdots ,1)}<f\left(x\right){|}_{x=(2,2,1,\cdots ,1)}$. From Lemma 3, vector (3,1,1,⋯,1) is associated with the leftmost node at level 3. So, the base case is true.

Induction hypothesis: Let, at level h=k(k>3), cost of f(x) for vector (k,1,⋯,1) (which is the leftmost node at level *k* according to Lemma 3) is $W_{k}^{\prime }$. Now, we choose any arbitrary node at level *k* which is different from the leftmost node and consider its associated cost as $W_{k}^{\prime \prime }$. Let us assume, $W_{k}^{\prime }<W_{k}^{\prime \prime }$.

Inductive step: If we choose the cost associated with the leftmost node at level k+1 as $W_{k+1}^{\prime }$, then from Lemma 3 and the tree construction rule we get,

(13) $$\begin{array}{c} \;W_{k+1}^{\prime }=\frac{q-x^{\prime }-1}{q-x^{\prime }}\;W_{k}^{\prime }\\ \mathrm{or},\;\;W_{k}^{\prime }=\frac{q-k}{q-k-1}\;W_{k+1}^{\prime } \end{array}$$

Now, we have to choose an arbitrary node at level k+1 which is different from the leftmost node. Without any loss of generality, here we choose a node which is a child of the arbitrarily chosen node (with cost $W_{k}^{\prime \prime }$) at the induction hypothesis section. Let, the cost of the selected child node at level k+1 is $W_{k+1}^{\prime \prime }$. The relation between $W_{k}^{\prime \prime }$ and $W_{k+1}^{\prime \prime }$ is,

(14) $$W_{k}^{\prime \prime }=\frac{q-x^{\prime \prime }}{q-x^{\prime \prime }-1}\;W_{k+1}^{\prime \prime }$$

where, $x^{\prime \prime }<k$ according to the tree construction rule. From the induction hypothesis,

(15) $$\begin{array}{c} \;W_{k}^{\prime }<W_{k}^{\prime \prime }\\ \;\mathrm{or},\;\;\frac{q-k}{q-k-1}\;W_{k+1}^{\prime }\;=\;\frac{q-x^{\prime \prime }}{q-x^{\prime \prime }-1}\;W_{k+1}^{\prime \prime }\\ \;\mathrm{or},\;\;\frac{W_{k+1}^{\prime }}{W_{k+1}^{\prime \prime }}\;=\;\frac{q^{2}-\left(k+x^{\prime \prime }+1\right)q+kx^{\prime \prime }+x^{\prime \prime }}{q^{2}-\left(k+x^{\prime \prime }+1\right)q+kx^{\prime \prime }+k}<1\\ \mathrm{or},\;\;W_{k+1}^{\prime }<W_{k+1}^{\prime \prime } \end{array}$$

 □

**Theorem** **1.** *The node with the minimum cost of the objective function, $f\left(x\right)=\prod_{i=1}^{M}\left(q-x_{i}\right)$ with respect to the constraint, $\sum_{i=1}^{M}x_{i}\;\leq N$ corresponds to the vector $\tilde{x}=\left(N-M+1,1,1,\cdots ,1\right)$, where q is the field size, m=M+1= generation size, n=N= number of receivers, $x=(x_{1},x_{2},\cdots ,x_{M})$, $\mathrm{and}\;x_{i}\in \left\{1,2,\cdots ,N\;-\;M\;+\;1\right\}\;\;\mathrm{for}\;i\in \left\{1,2,\cdots ,M\right\}$.*

**Proof.**  We can prove the theorem using Lemma 3, Propositions 1 and 2. □

**Corollary** **1.** *The set of optimal points of the nonlinear integer program in (10) is given as:*

(16) $$Q=\{x^{*}|x^{*}\;\mathrm{is}\;a\;\mathrm{vector}\;\mathrm{obtained}\;\mathrm{by}\;\mathrm{permuting}\;\mathrm{the}\;\mathrm{elements}\;\mathrm{of}\;\mathrm{the}\;\mathrm{vector}\;\tilde{x}=\left(N-M+1,1,\cdots ,1\right)\}$$

**Proof.**  This follows from Theorem 1 and the idea of *equivalent* vectors. □

With the help of Corollary 1 and (9), we get the minimum value of $P_{LL}$ as:

(17) $$\begin{array}{c} {\left.P_{LL}\right|}_{\min}=\frac{{\left(q-1\right)}^{m-2}}{q^{m-1}}\left(q-n+m-2\right) \end{array}$$

While solving the nonlinear program in (9), we have considered there is no such receiver which has not received any packet or which has received all the packets of the generation. This is to ensure that we obtain the lowest possible value of $P_{LL}$ which is given in (17). If there are |R(1)| receivers who have not received any packet and |E(m)| receivers who have received every packet, the objective function of the integer program will remain same as in (9), but the constraint will be changed to, $\sum_{i=2}^{m}|S\left(i\right)|\;\leq n-\left|R\left(1\right)\right|-\left|E\left(m\right)\right|$. Here, we will also be able to find the minimum value of the objective function in the same manner described above, but this value will be greater than the value obtained in (17). So, we conclude that (17) provides the absolute minimum value of $P_{LL}$.

**Figure 6:**
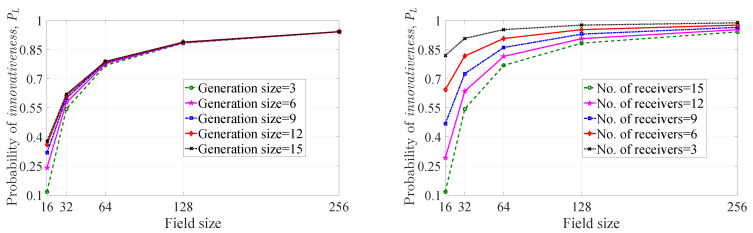
Minimum value of the lower bound of the probability of *innovativeness* (${\left.P_{LL}\right|}_{\min}$) of a picked-up linear combination versus field size. For the left subfigure plots, we have considered n=15 and m=3, m=6, m=9, m=12, m=15, and for the right subfigure plots, we have considered m=3 and n=3, n=6, n=9, n=12 and n=15.

We plot the minimum value of the lower bound $\left(P_{LL}\right)$ according to (17) in Figure 6 with respect to field size. In the left subfigure, the total number of receivers (*n*) is fixed at 15 while we vary the generation size (*m*) from 3 to 15 with an interval of 3. Similarly, in the right subfigure, *m* is fixed at 3, and *n* is changed from 3 to 15 with interval 3. The left graph shows that for a fixed group of receivers the lower bound gets improved (though minimal improvement) with the increment of generation size for a fixed field. So, larger generation size can be beneficial from the $P_{LL}$ perspective. But, a large generation size will increase the encoding complexity. Hence, generation size optimization is required according to the application where rDWS scheme is incorporated. In contrast, the right graph shows that the lower bound gets deteriorated (significant deterioration) with the increase of the number of receivers. This happens because the $c_{j}\left(i\right)$ heterogeneity increases with the number of receivers.

Secondly, the minimum value of the lower bound $\left(P_{LL}\right)$ is close to one for each plot of both graphs at field size 256. So, the chance of getting an *innovative* combination for rDWS encoding approaches certainty with the increase of field size. In general, this is true for any generation size and any number of receivers. So we conclude that, apart from the computational complexity benefit of our proposed rDWS technique, its encoding achieves near-optimal performance for a finite field of sufficient size. This fact also ascertains that the rDWS encoding is identical to the conventional random linear network encoding [7].

#### A Practical Consideration and the Minimum Value of $P_{LL}$

In Section 4.1, we have seen that R(i)=T(i)∪S(i) (except for i=1 where R(1)=T(1)). There it was argued that the probability of *innovativeness* is always one with respect to any receiver of T(i). But, if the CM chooses the zero element of the field under consideration as $\gamma_{i}$, the receivers of T(i) will fail in *seeing* $p_{i}$ upon successful reception of the coded combination. So practically, the number of available *good choices* with respect to any receiver of T(i) is q−1.

For i≠1, let us look at $P_{C}\left(i\right)$ for the following considerations:

**Case** **1.** *When |R(i)|=|S(i)| (i.e., |T(i)|=0), we get $P_{C}\left(i\right)\geq \frac{q-|R(i)|}{q}$ (from (5)).*

**Case** **2.** *Next, let |T(i)|=1 and |S(i)|=|R(i)|−1. In the worst situation, all receivers in S(i) have different $c_{j}\left(i\right)$’s in their corresponding $y_{j}$ equation and none of them are zero. The CM must not choose any of these $c_{j}\left(i\right)$’s (because of the receivers in S(i)) or the zero element of $F_{q}$ (because of the receiver in T(i)) as $\gamma_{i}$. Hence, the total number of available good choices with respect to the receivers in R(i) is (q−(|R(i)|−1)−1)=q−|R(i)|. Subsequently, we get $P_{C}\left(i\right)\geq \frac{q-|R(i)|}{q}$.*

**Case** **3.** *Next, we consider |T(i)|=2 and |S(i)|=|R(i)|−2. With similar argument given in case 2, we get $P_{C}\left(i\right)\geq \frac{q-|R(i)|+1}{q}$.*

If we proceed in this way, as the terminal case we obtain |R(i)|=|T(i)| (i.e., |S(i)|=0). Here, it is evident that $P_{C}\left(i\right)=\frac{q-1}{q}$.

Summarizing the above discussion it can be concluded that, for i≠1, the lower bound on the probability of *innovativeness* of a coefficient is $P_{CL}^{\prime }\left(i\right)=\frac{q-|R(i)|}{q}$, whereas for i=1, the probability of *innovativeness* is $P_{C}^{\prime }\left(1\right)=\frac{q-1}{q}$. Using these findings, we obtain a lower bound on the probability of *innovativeness* of the linear combination as:

(18) $$P_{LL}^{\prime }=\frac{q-1}{q^{m}}\prod_{i=2}^{m}\left\{q-|R\left(i\right)|\right\}$$

Our next goal is to search for the minimum value of $P_{LL}^{\prime }$. Likewise in Section 4.2.1, here the minimum value can be obtained by solving the following nonlinear integer programming:

(19) $$\begin{array}{c} \;\mathrm{Minimize}:\;\frac{q-1}{q^{m}}\prod_{i=2}^{m}\left\{q-|R\left(i\right)|\right\}\\ \mathrm{Subject}\;\mathrm{to}:\;\sum_{i=2}^{m}|R\left(i\right)|\;=n-\left|E\left(m\right)\right|-\left|T\left(1\right)\right| \end{array}$$

Following the method of solution presented in Section 4.2.1, we get the minimum value of the lower bound as $\frac{{\left(q-1\right)}^{m-1}\left(q-n+m+|E\left(m\right)|+|T\left(1\right)|-2\right)}{q^{m}}$. One can infer that the absolute minimum value of $P_{LL}^{\prime }$ is obtained when |E(m)|=0 and |T(1)|=1 (as |T(1)| can not be zero) which we write as:

(20) $${\left.P_{LL}^{\prime }\right|}_{\min}=\frac{{\left(q-1\right)}^{m-1}}{q^{m}}\left(q-n+m-1\right)$$

**Figure 7:**
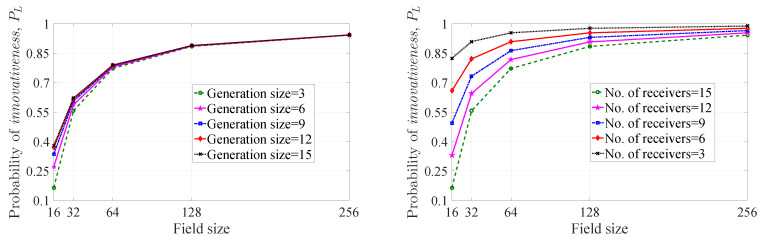
Minimum value of the lower bound of the probability of *innovativeness* (${\left.P_{LL}^{\prime }\right|}_{\min}$) of a picked-up linear combination versus field size. For the left subfigure plots, we have considered n=15 and m=3, m=6, m=9, m=12, m=15, and for the right subfigure plots, we have considered m=3 and n=3, n=6, n=9, n=12 and n=15.

In Figure 7, we plot the minimum value of the lower bound $P_{LL}^{\prime }$ according to (20) in the same manner as we did for ${\left.P_{LL}\right|}_{\min}$ in Section 4.2.1. The results of Figure 6 and Figure 7 are very similar as there is not much difference in the values of ${\left.P_{LL}\right|}_{\min}$ and ${\left.P_{LL}^{\prime }\right|}_{\min}$. So, similar conclusions can be drawn from Figure 7 as we have discussed for Figure 6 at the end of Section 4.2.1.

## 5. Performance Evaluation of rDWS Scheme in Terms of Dropping and Decoding Statistics

So far, we have discussed the rDWS algorithms and analyzed the throughput efficiency of the scheme to compare it to the state of the art. As mentioned previously, while developing the rDWS technique it is ensured that, the sender does not necessarily lose its packet dropping capability even before decoding at receivers like DWS technique. The modified QUM algorithm ensures that. In this section, we evaluate the statistical performance of packet dropping and packet decoding of our proposed scheme by means of simulation.

We create a *Monte Carlo* simulation environment where the model described in Section 2 is implemented in a discrete time, single transmitter, multiple receiver broadcasting scenario with packet erasure channels. The channels are independent and offer homogeneous, Bernoulli packet loss. ARQ is realized from the receivers to the transmitter through perfect, error-free feedback channels. As said before, packets are delivered (which also implies packets are *seen*) to the receivers in the same order they arrive at the SQ. So, packet $p_{i}$ is *seen* before $p_{i+1}$. While doing the simulations, we assume negligible propagation delay and negligible processing time for various operations (like encoding at the sender, ACK/NACK generation at the receivers etc.) compared to a time slot.

The metrics which we consider for the statistical performance evaluation of the rDWS technique are the cumulative packet dropping probability, the average time to drop the last packet of a generation at the sender, and the cumulative packet decoding probability at the receivers. These metrics for rDWS depend on the size of the field, which is used to form the coded combinations. We consider the extension fields of GF(2) and perform the simulations with increasing field size. Finally, the DWS technique is simulated with the same setup, and the results are compared with the rDWS counterparts to get a comprehensive picture of the performance of the proposed approach. For the DWS consideration, the corresponding finite field is also chosen to be an extension field of GF(2) with the constraint that the field size is greater than or equal to the number of receivers. While doing the throughput efficiency analysis of the rDWS technique, we have also restricted our attention only to the extension fields of GF(2) whose size is greater than or equal to the number of receivers. But, here we relax that constraint for two basic reasons. First one is to observe how rDWS-based broadcast performs with small finite fields. Second reason is to show the usefulness of the rDWS over DWS as the later technique must not be performed with a finite field of size lesser than the number of receivers.

**Figure 8:**
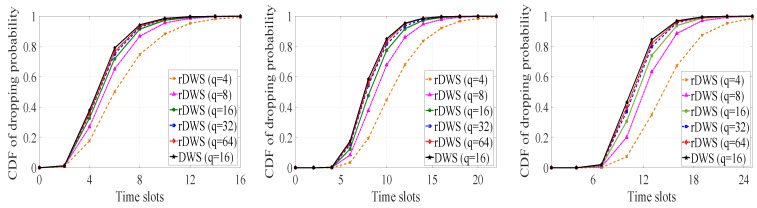
Cumulative probability of packet dropping for the first packet (left subfigure), the second packet (middle subfigure), and the third packet (right subfigure) versus time slots with n=15, m=3 and $p_{e}=0.5$.

**Figure 9:**
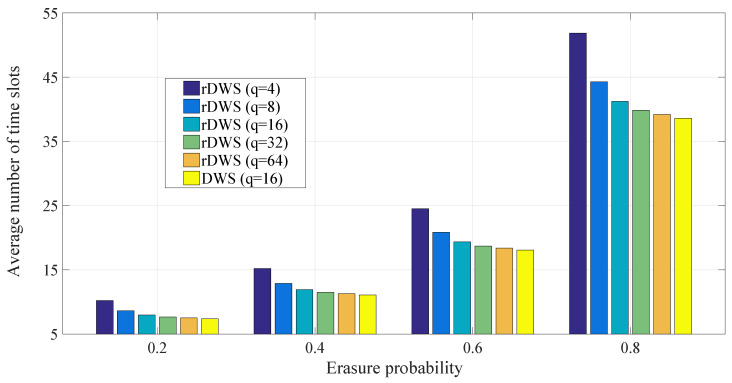
Average time to drop the last packet of a generation versus channel erasure probability with n=15 and m=4.

**Figure 10:**
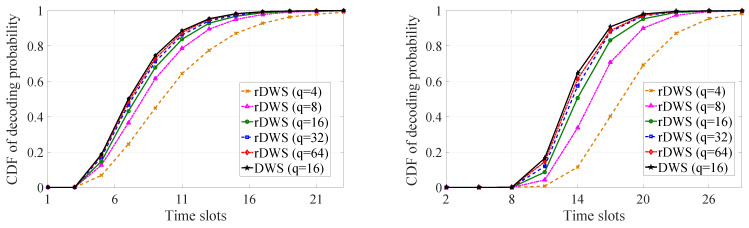
Cumulative probability of decoding a generation for an arbitrarily chosen receiver (left subfigure), and for the whole receiver system (right subfigure) with n=15, m=4 and $p_{e}=0.5$.

We conduct $10^{5}$ independent runs for each evaluation scenario. The results are averaged over all runs and are plotted in the respective figures from Figure 8, Figure 9 and Figure 10.

### 5.1. Packet Dropping Statistics

The QUM drops a packet from SQ when all receivers have *seen* it. At an arbitrary time slot *t*, let the state of a receiver is the index of its last *seen* packet. Clearly, the QUM is in a position to drop the packet with index same as the minimum state of all receivers at *t*.

#### 5.1.1. Cumulative Dropping Probability

First, we look at the dropping probability of the packets of a generation. For 15 broadcasting receivers and erasure probability 0.5, we plot the cumulative dropping probability (with respect to time) of the packets of a generation with generation size 3 in Figure 8. Here, the cumulative probability of dropping a packet at *t* implies the total probability of dropping that packet from slot 1 to slot *t*. As there is no actual field size requirement for rDWS technique (Section 3.1.2), we perform the simulations for the respective fields: GF($2^{2}$), GF($2^{3}$), GF($2^{4}$), GF($2^{5}$) and GF($2^{6}$). Finally, the cumulative dropping probability is plotted for DWS case where the field, GF($2^{4}$) is considered (as total number of receivers is 15).

Figure 8 consists of three subfigures. From left to right, the subfigures correspond to the cumulative probability for the first, second and third packet of the generation respectively. For each subfigure, at a particular time slot, the dropping probability for rDWS cases increases and gradually becomes closer to the same for DWS case with the increase of field size. The CM of the rDWS technique picks random coefficients for the linear combination, and the chance of getting *innovative* coefficients increases with increasing field size. As a consequence, the chance of state transition for a receiver increases which in turn enhances the packet dropping probability. From these observations, it is clear that the dropping probability for rDWS case is going to be very close to the same of the DWS case for a field of sufficiently large size, and that size directly depends on the broadcast settings. Here in our plots, one can observe that the dropping probability with respect to GF($2^{6}$) for rDWS case is in fact very close to the DWS case at each time slot (in every subfigure).

In Figure 8, the dropping probability curve for a particular rDWS case (or for the DWS case) grows at a relatively slower rate towards unity as we move from the left to the right subfigure. This is the result of the in-order delivery assumption. Packets are *seen* by the receivers in the same order they arrive at SQ, and the cumulative probability grows at a slower pace from the first to the third packet.

#### 5.1.2. Average Dropping Time for the Last Packet of a Generation

Next, we evaluate the average number of time slots required to drop the last packet of a generation and plot these values with respect to erasure probability in Figure 9. Because of the in-order packet delivery, dropping the last packet implies dropping the whole generation. So, the average dropping time for the last packet is essentially identical to the average time to evacuate the generation from SQ.

The simulations are performed for 15 broadcasting receivers and generation size four. We plot the average number of time slots for four erasure probabilities, 0.2, 0.4, 0.6 and 0.8. In each erasure probability case, rDWS simulations are done for fields, GF($2^{2}$), GF($2^{3}$), GF($2^{4}$), GF($2^{5}$) and GF($2^{6}$), and DWS simulation is done for GF($2^{4}$). Though the erasure probability is increased with an interval of 0.2 starting from 0.2, the average number of slots to drop the generation does not increase in an uniform manner. As an example, for rDWS with field GF($2^{2}$), the average number of slots are 10, 15, 25 and 52 (approximate values) respectively. This is happening due to the rapid increase of erasure heterogeneity at a time slot with erasure probability. Though the channels are homogeneous in nature from erasure probability perspective, at a particular slot, whether a channel is in erasure or not is independent from the other channels. This phenomenon brings the slot-wise erasure heterogeneity for the channels which is increased in a non-uniform and rapid manner with the increase of erasure probability.

For each erasure probability consideration, the average number of slots for rDWS tends towards the DWS case as we move from GF($2^{2}$) to GF($2^{6}$). One can argue similarly as in Section 5.1.1 and conclude that DWS refers to the limiting behavior of the rDWS cases with increasing field size. In our evaluation, the performance of the rDWS scheme with GF($2^{6}$) is sufficiently close to the DWS scenario. One more point to notice from Figure 9 is: the plots (all five plots for rDWS and the DWS plot) get sparse from each other as we increase the erasure probability. This is also due to the non-uniform and rapid increase of slot-wise erasure heterogeneity with erasure probability.

Now, the sender is able to drop the last packet of the generation indicates that all receivers have *seen* all packets of the generation, and the generation can be decoded. Thus, the generation size divided by the average time to drop the last packet of the generation provides the throughput of the whole broadcasting system. For instance, throughput of the rDWS broadcasting system (from Figure 9) with GF($2^{2}$) for erasure probability 0.8 is 4/52=0.077 packets/ slot (approximate value). So, Figure 9 provides an indirect measure of the throughput of the systems under consideration. Higher the average number of slots, lesser the throughput is.

### 5.2. Generation Decoding Statistics

While broadcasting the packets of a generation, the *knowledge space* of the transmitter is considered to be of dimension *m*. When a receiver *sees* a packet, the dimension of its *knowledge space* increases by one. The receiver can decode the generation when the dimension of its *knowledge space* becomes equal to the same of the transmitter’s i.e., the *knowledge space* matrix (Section 3.2) of that receiver attains full-rank.

In this section, we investigate the cumulative probability of decoding a generation in two different contexts. In the first context, we find the decoding probability for an arbitrarily chosen receiver. Secondly, we find the same for the whole receiver system. Obviously, decoding of a generation by the receiver system is equivalent to decoding by the receiver (or receivers) who is the last receiver to *see* packet $p_{m}$.

We plot the cumulative decoding probability of a generation with respect to time in Figure 10, for a generation of size four. Total 15 broadcasting receivers are considered as before, and the erasure probability is 0.5. In the left subfigure, decoding probability for an arbitrary single receiver is depicted, whereas in the right subfigure, decoding probability for the whole receiver system is plotted. For both subfigures, the simulations are performed for five instances of the rDWS case and for the DWS case (same as in Section 5.1), and the results are plotted in each case.

For a fixed rDWS case (or for the DWS case), the cumulative probability curve for single receiver grows faster than the curve for all receiver in Figure 10. This is evident as decoding by all receivers is equivalent to decoding by the last receiver in the list. In both of the subfigures, the performance of the rDWS scheme with GF($2^{6}$) is very close to the performance of the DWS technique.

## 6. Conclusions

In this paper, we have proposed a randomized version of the existing DWS technique for network coded broadcast. The main goal of this modified technique is to reduce the computational complexity of the existing algorithms while keeping the essence of the original technique intact. So, the notion of *seeing* a packet and the transmitter’s ability to drop a packet before decoding at receivers are also present in our rDWS scheme. We exploit the fact that the receivers always keep track of their own *knowledge space*. Instead of keeping each receiver’s complete *knowledge space* information, the transmitter gathers the dimension of the spaces through feedback (in the form of NACK). Implementation of this logic leads to much simpler and computationally less expensive algorithms. But, the price of this achievement is paid in the form of non-optimal encoding. From throughput efficiency analysis of the rDWS technique, we obtain a lower bound on the *innovativeness* probability of the coded combination with the consideration that all packets of the generation are used in the linear combination. Our theoretical and numerical performance analyses imply that rDWS achieves throughput optimality in an asymptotic sense with the increase of field size.

When we have a strong processor with large enough memory, DWS offers required performance though it involves high computational complexity. However, as future generation wireless technology might require lightweight, fast, and simple algorithms for packet processing, rDWS might just turn out to be a better choice. One can observe that the possible increment in number of retransmissions and consequent increase in latency with rDWS technique can easily be compensated with larger field size (Figure 8, Figure 9 and Figure 10). As it is demonstrated in Figure 9, for an erasure probability 0.2, even with a smaller field of size 8, rDWS exhibits only 16.51% larger average latency than the same of the DWS with field size 16. If we allow an equal field size, the difference gets reduced to 7.78%, and with a larger field of size 32 for rDWS, it gets reduced to just 3.81%. For an erasure probability 0.8, with a smaller field of size 8, rDWS exhibits only 14.76% larger average latency than the same of the DWS with field size 16. If we allow an equal field size, the difference gets reduced to 6.85%, and with a larger field of size 32 for rDWS, it gets reduced to just 3.21%. So, with realistic and moderate to even bad channel conditions, one can have an almost optimal performance with proposed lightweight, low complexity rDWS algorithms. A large field and generation size is relevant to desktop-based applications, whereas embedded platforms and high-end smartphone-based applications require low to moderate field and generation size [65]. Most of the security-related operations require high generation and field size, but video streaming and P2P file sharing require low to moderate values of field and generation size [65]. It is assumed that a low to moderate field and generation size will lead to low complexity algorithms which are compatible with next-generation wireless technology vision and requirements. Here, rDWS can be the candidate of choice as the same, while remaining a low complexity technique and using field sizes same to that of the DWS, provides near-optimal (almost as good as DWS) performance at moderate to difficult channel conditions.

As an immediate future extension of this work, a thorough mathematical analysis of the packet dropping and decoding statistics of the rDWS scheme can be done. A similar analysis for DWS technique was performed in [61]. Though we have carried out the same statistical analysis in Section 5 through simulation, a mathematical analysis of the same will help in getting a strong theoretical underpinning about the performance of the rDWS scheme.

Generation-based network coding is less suitable for real-time broadcast. So, another challenge is to search for a modified version of the rDWS scheme which will be ideal for real-time broadcasting system.

As the third extension of our work, a queueing theoretic analysis can be done with a stochastic arrival to analyze the issues related to the SQ stability.

## Figures and Tables

**Table 1:** The probability of *innovativeness* for all possible partitions of |S(3)| when |S(3)|=4.

Form of c(3)	Partition of |S(3)|=4	No. of Parts	No. of *Good* *Choices* for $\gamma_{3}$	Probability of *Innovativeness*
$\left(\alpha_{1},\alpha_{2},\alpha_{3},\alpha_{4}\right)$	1 + 1 + 1 + 1	4	q−4	$\frac{q-4}{q}$
$\left(\alpha_{1},\alpha_{2},\alpha_{3},\alpha_{3}\right)$	1 + 1 + 2	3	q−3	$\frac{q-3}{q}$
$\left(\alpha_{1},\alpha_{1},\alpha_{2},\alpha_{2}\right)$	2 + 2	2	q−2	$\frac{q-2}{q}$
$\left(\alpha_{1},\alpha_{2},\alpha_{2},\alpha_{2}\right)$	1 + 3	2	q−2	$\frac{q-2}{q}$
$\left(\alpha_{1},\alpha_{1},\alpha_{1},\alpha_{1}\right)$	4	1	q−1	$\frac{q-1}{q}$

## Abbreviations

The following abbreviations are used in this manuscript:

LNC	Linear network coding
RLNC	Random linear network coding
DLNC	Deterministic linear network coding
IDNC	Instantly decodable network coding
SQ	Sender queue
DWD	Drop when decoded
DWS	Drop when seen
rDWS	Randomized drop when seen
QUM	Queue update module
CM	Coding module
